# A hypothalamic circuit underlying the dynamic control of social homeostasis

**DOI:** 10.1038/s41586-025-08617-8

**Published:** 2025-02-26

**Authors:** Ding Liu, Mostafizur Rahman, Autumn Johnson, Ryunosuke Amo, Iku Tsutsui-Kimura, Zuri A. Sullivan, Nicolai Pena, Mustafa Talay, Brandon L. Logeman, Samantha Finkbeiner, Lechen Qian, Seungwon Choi, Athena Capo-Battaglia, Ishmail Abdus-Saboor, David D. Ginty, Naoshige Uchida, Mitsuko Watabe-Uchida, Catherine Dulac

**Affiliations:** 1 Department of Molecular and Cellular Biology, Howard Hughes Medical Institute, Center for Brain Science, Harvard University, Cambridge, MA USA; 2 Department of Molecular and Cellular Biology, Center for Brain Science, Harvard University, Cambridge, MA USA; 3 Department of Neurobiology, Howard Hughes Medical Institute, Harvard Medical School, Boston, MA USA; 4 Department of Biological Sciences, Zuckerman Mind Brain Behavior Institute, Columbia University, New York, NY USA; 5 Present Address: Division of Brain Sciences, Institute for Advanced Medical Research, Keio University School of Medicine, Tokyo, Japan; 6 Present Address: Department of Psychiatry, UT Southwestern Medical Center, Dallas, TX USA

**Keywords:** Social behaviour, Neural circuits

## Abstract

Social grouping increases survival in many species, including humans^1,2^. By contrast, social isolation generates an aversive state (‘loneliness’) that motivates social seeking and heightens social interaction upon reunion^3–5^. The observed rebound in social interaction triggered by isolation suggests a homeostatic process underlying the control of social need, similar to physiological drives such as hunger, thirst or sleep^3,6^. In this study, we assessed social responses in several mouse strains, among which FVB/NJ mice emerged as highly, and C57BL/6J mice as moderately, sensitive to social isolation. Using both strains, we uncovered two previously uncharacterized neuronal populations in the hypothalamic preoptic nucleus that are activated during either social isolation or social rebound and orchestrate the behaviour display of social need and social satiety, respectively. We identified direct connectivity between these two populations and with brain areas associated with social behaviour, emotional state, reward and physiological needs and showed that mice require touch to assess the presence of others and fulfil their social need. These data show a brain-wide neural system underlying social homeostasis and provide significant mechanistic insights into the nature and function of circuits controlling instinctive social need and for the understanding of healthy and diseased brain states associated with social context.

## Abstract (web summary)

New data on brain-wide circuits centred around two interconnected hypothalamic neuron populations provide significant mechanistic insights into the emergence of social need during social isolation and the satiation of social need during social reunion.

## Main

Humans readily form groups and flourish in social activities, whereas prolonged social isolation leads to increased anxiety, fragmented sleep, impaired cognition, weakened immune system and increased risk for cardiovascular illnesses and cancer^7,8^. Similarly, in many animal species, social isolation leads to abnormal behaviours and increased disease susceptibility^5,9,10^, whereas grouping decreases risks of predation and energy consumption, and enables collaborative behaviours such as parenting and group foraging^1,2^. The aversive state of social isolation may thus reveal an evolutionarily conserved alarm signal that promotes social seeking^6,8^.

Essential physiological needs such as those for food, water and sleep are encoded by conserved brain circuits that monitor the organism’s internal state and drive goal-directed behaviours—eating, drinking or sleep that restore homeostasis. The intensity of the restoring behaviour matches the amount of deprivation, leading to satiation when the organism’s needs are fulfilled^11^. The hypothalamus has emerged as a brain hub underlying physiological homeostasis, where specific neuronal populations orchestrate distinct survival needs. Examples include neurons in the arcuate nucleus (Arc) expressing *AgRP* and *POMC* for the control of hunger and food satiety, respectively^12,13^, neurons in lamina terminalis and MnPO for water intake^14–16^ and several populations for sleep control^17^. Increased durations of social isolation in rodents trigger stronger rebounds in social interaction, indicating a homeostatic process elicited by social deprivation in a manner similar to that of other physiological needs^3,4,6^.

Recent studies have identified the roles of dopamine-, oxytocin- and serotonin-associated brain circuits in mediating social motivation and reward upon reunion^18–20^. However, the neural mechanisms underlying social need elicited by isolation and social satiation following reunion remain poorly defined. Here, we proposed that specific hypothalamic circuits may exist that underlie social homeostasis.

We uncovered two genetically defined populations of neurons in the mouse hypothalamus and associated brain-wide circuits that orchestrate the regulation of social need and social satiety, and identified touch as a critical sensory modality informing mice about their social context. Our findings provide significant insights into the neural basis of instinctive social drive and may provide new avenues to the understanding of social behaviour in normal and pathological contexts.

## Social rebound reflects social homeostasis

**Fig. 1:**
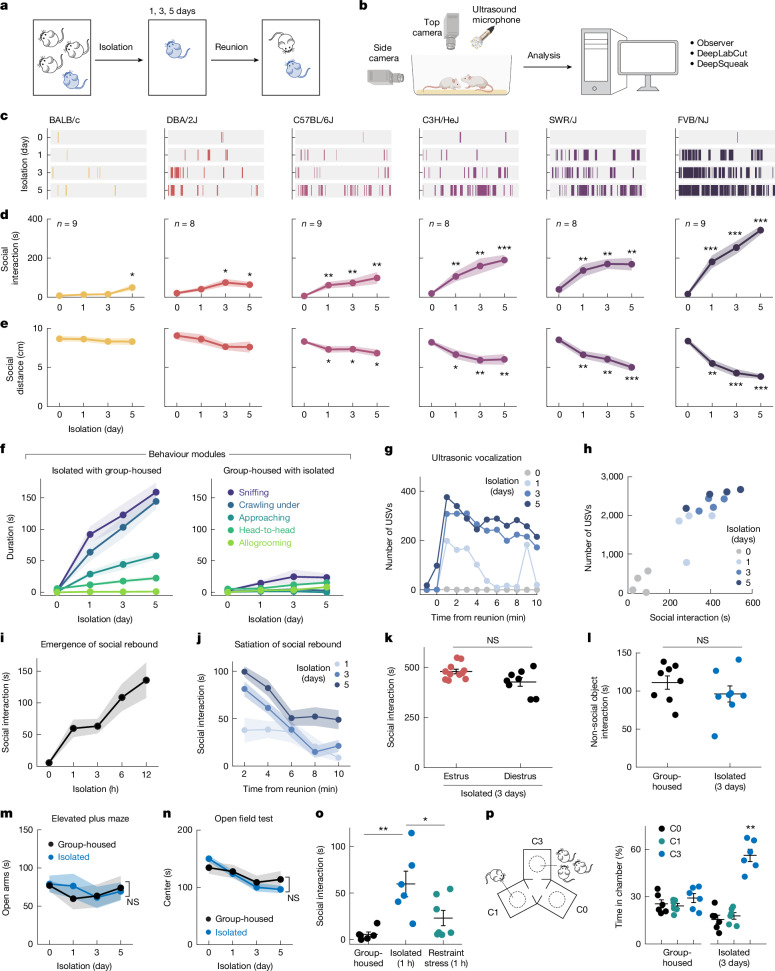
Social rebound as behaviour manifestation of social homeostasis. **a**,**b**, Short-term social isolation/reunion behavioural paradigm (**a**) and analysis pipeline (**b**). **c**, Raster plots of social events during reunion for an example mouse from each strain. Isolation day 0 refers to group housing condition. **d**,**e**, Total duration of social interaction (**d**) and average social distance (**e**) between two mice during a 10-min social reunion. **f**, Behavioural modules displayed during social reunion by previously isolated (left) versus group-housed FVB mice (right); *n* = 7. **g**, USVs during social reunion from an example FVB mouse. **h**, Correlation between duration of social interaction and number of USV syllables during social reunion. Each dot represents a mouse; *n* = 4. **i**, Emergence of social rebound in FVB mice following increasing duration of isolation; *n* = 6. **j**, Satiation of social rebound after various lengths of isolation; *n* = 7. **k**, Social rebound in different phases of the oestrous cycle: oestrus phase *n* = 11; dioestrus phase, *n* = 8. **l**, Investigation of non-social object in group-housed versus isolated FVB mice; *n* = 8. **m**,**n**, Elevated plus maze test (**m**; *n* = 5) and open field test (**n**; *n* = 9) in isolated and group-housed FVB mice. **o**, Social interaction in FVB mice after 1 h of social isolation (*n* = 6) or body restraint stress (*n* = 7). **p**, Preference test among empty (C0), one-mouse (C1) and three-mice (C3) chambers; *n* = 6. Diagram in **b** was created using BioRender.com. **d**,**e**, Friedman test between baseline (day 0) and each isolation day; **k**,**l**,**o**, Mann–Whitney *U-*test; **m**,**n**, Two-way analysis of variance; **p**, Friedman test. NS, not significant; *P < 0.05, **P < 0.01, ***P < 0.001. All shaded areas and error bars represent the mean ± s.e.m.

**Extended Data Fig. 1:**
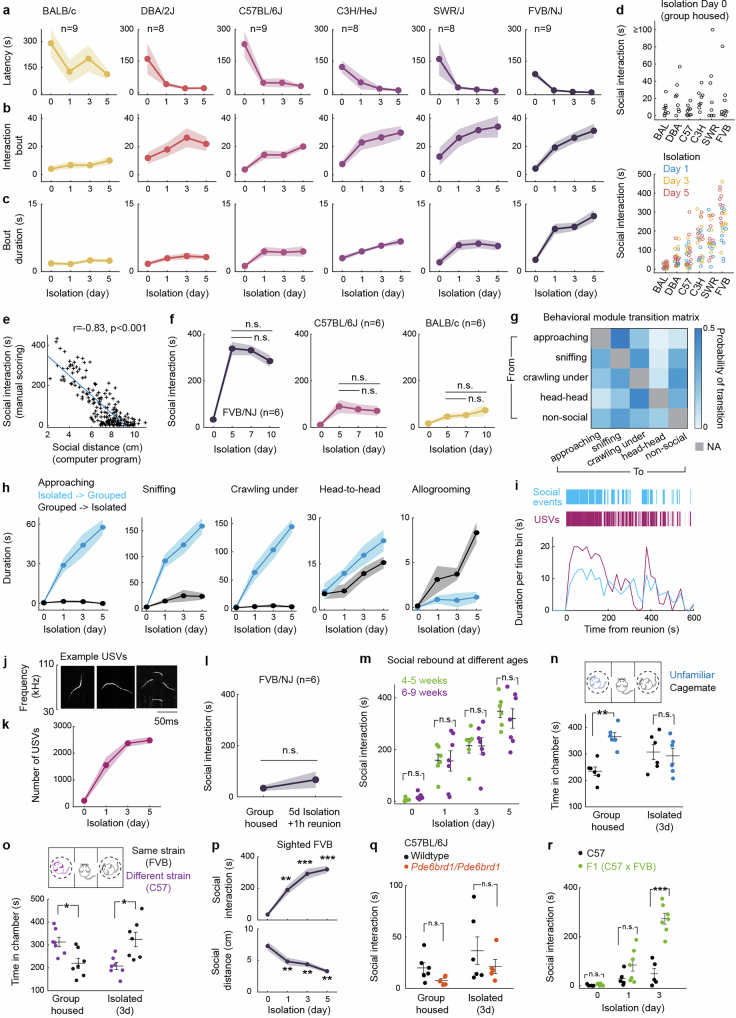
Behavioral characterization of social rebound. **a-c**, Latency, bout number and mean bout duration of social interactions during social reunion (BALB/c n = 9, DBA/2 J n = 8, C57BL/6 J n = 9, C3H/HeJ n = 8, SWR/J n = 8, FVB/NJ n = 9). **d**, High magnification plots of social interaction durations (Fig. 1d) for individual mice from all tested strains. **e**, Correlation between manually scored and computer program calculated social interactions. Each + represents a mouse per reunion test. n = 198. Pearson correlation analysis. **f**, Social interaction in three mouse strains after more than 5 days of isolation. n = 6 mice. **g**, Probability matrix of transitions between behavioral modules during social rebound in FVB mice, n = 7. **h**, Display of specific behavioral modules in paired isolated (blue) vs group housed (black) FVB mice, n = 7. **i**, Time course of social events and USVs from an example FVB mouse. **j**, Example ultrasonic vocalization (USV) syllables of FVB mice recorded during social reunion. **k**, Number of USV syllables during social reunion in FVB mice after various lengths of isolation, n = 4. **l**, Social need satiation after 1 h of social reunion in FVB mice, n = 6. **m**, Social rebound in FVB mice of different ages, 4-5 weeks, n = 6; 6–9 weeks, n = 7. **n**, Three-chamber preference test between a cagemate and a stranger, in group housed vs isolated FVB mice, n = 6. **o**, Preference test between mice from the same (FVB) or different (C57) strains, n = 7. **p**, Behavioral responses to social isolation in the sighted FVB mice, n = 6. **q**, Social interactions measured in wildtype (n = 6) versus blind (n = 5) C57 mice. **r**, Social interactions in C57 versus the offspring of FVB/C57 cross, n = 5-7. **f**, **p**, Friedman test; **l**, **n**, **o**, Wilcoxon signed-rank tests; **m**, **q**, **r**, Mann–Whitney U test; n.s., not significant; *p < 0.05, **p < 0.01, ***p < 0.001. All shaded areas and error bars represent the mean ± s.e.m.

Leveraging the preference of laboratory mice for grouped over isolated housing^18,21^, we separated adult sibling cagemates, housed them singly for up to 5 days and quantified the subsequent rebound in social interaction during reunion as a proxy for the underlying social need (Fig. 1a,b). Experiments were performed in adult females to avoid interfering behaviours (aggression or mating) seen in adult males. We assessed the amount of social need in six mouse strains and observed significant social rebounds in all strains (Fig. 1c–e). The strength of social rebound, measured as total interaction time, interaction bout number and bout duration, increased steadily, whereas distance between animals and behavioural latency decreased with longer isolation (Fig. 1c–e and Extended Data Fig. 1a–e), providing a measure of the increased social need elicited by lengthier isolation. Different strains showed highly diverse ranges of social rebound, from weak (BALB/c and DBA) to moderate (C57BL/6J, C3H/HeJ, SWR/J) and strong (FVB/NJ), indicating distinct sensitivities to social isolation according to genetic background (Fig. 1c–e and Extended Data Fig. 1a–d). The strength of rebound in each strain was unrelated to the reported stress sensitivity for that strain^22,23^. No further increase in social rebound was observed past day 5 (Extended Data Fig. 1f), indicating limits in accumulation of social need, also observed during long-term isolation^6,9^. On the basis of these data, we selected the mouse strains FVB/NJ (FVB) and C57BL/6J (C57) for further behavioural and functional experiments.

Distinct behavioural modules and robust ultrasonic vocalizations (USVs) were identified during social rebound in FVB mice (Fig. 1f–h, Extended Data Fig. 1g–k and Supplementary Video 1), with behaviour events initiated mostly by the previously isolated mice (Fig. 1f and Extended Data Fig. 1h). Behaviour analysis following short isolation (1–12 h) showed the emergence of progressively more intense social rebound with increased isolation time (Fig. 1i). By contrast, rebound behaviour declined over time during reunion, indicating a gradual satiation of social need (Fig. 1j), with 1 h of reunion satiating the need accumulated by 5 days of isolation (Extended Data Fig. 1l). The social rebound was independent from the female oestrus stage (Fig. 1k) or age (Extended Data Fig. 1m) and was not associated with any general increase in object investigation (Fig. 1l) or anxiety level (Fig. 1m–o). Isolated mice, unlike group-housed mice, preferred to interact with a group over a single mouse (Fig. 1p) and showed reduced preference for new encounters (Extended Data Fig. 1n,o), supporting the idea that social isolation promotes an affiliative drive that compels animals back to their social group.

FVB mice suffer from progressive retinal degeneration owing to homozygosity of the *Pde6b*^*rd1*^ allele^24^. The FVB strain with restored vision (*Pde6b*^+/+^)^25^ showed similar amounts of social rebound as blind FVB mice (Extended Data Fig. 1p) and, conversely, C57 mice carrying the *Pde6b*^*rd1*^*/Pde6b*^*rd1*^ mutation showed no difference in rebound behaviour compared with C57 wild-type mice (Extended Data Fig. 1q). Moreover, *Pde6b*^*rd1/+*^ offspring of a FVB and C57 cross, which have functional vision^26^, exhibited significantly higher social rebound than the C57 strain (Extended Data Fig. 1r). Together, these data indicate that the elevated social rebound seen in FVB mice results from unique features in the genetic background of the FVB strain and not from impaired vision.

## Candidate neurons underlying social homeostasis

**Fig. 2:**
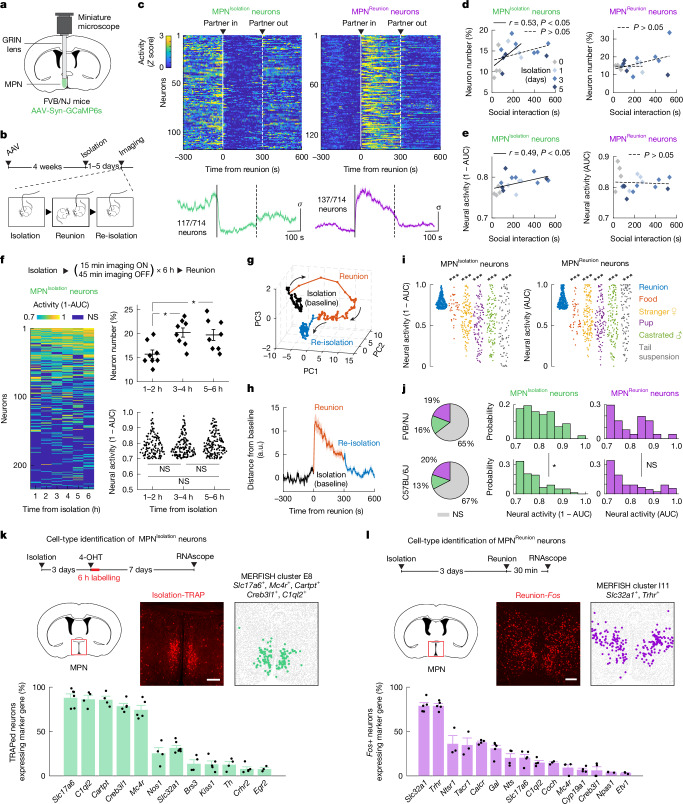
Identification of neuron types underlying social homeostasis. **a**, Microendoscopy calcium imaging in the hypothalamic medial preoptic nucleus (MPN). **b**, Behavioural and calcium imaging paradigm. **c**, Heatmap and average activity of MPN neurons activated during isolation or reunion in FVB mice after 3 days of isolation; *n* = 6 mice. **d**,**e**, Changes in number (**d**) and neural activity (**e**) of MPN^Isolation^ and MPN^Reunion^ neurons as a function of social rebound after various lengths of isolation in FVB mice; n = 4–6 mice for each isolation condition. Pearson correlation analysis. **f**, Activity analyses of MPN^Isolation^ neurons during the first 6 h of isolation; *n* = 4 mice; *P < 0.05; Friedman test. **g**, PCA of population activity in **c**. Bin size, 5 s. **h**, Distance from each point in the neural trajectory **g** to the mean of baseline; bin size, 1 s. **i**, Activity specificity analyses of MPN neurons during different behavioural conditions; *n* = 4 FVB mice. ***P < 0.001, paired *t*-tests of neuronal activity of the same neuron ensemble between reunion and another control condition. **j**, MPN neuronal activity after 3 days of isolation in the FVB and C57 mouse strains; *n* = 6 mice for each strain. *P < 0.05, two-sample Kolmogorov–Smirnov test. **k**,**l**, Cell-type identification of MPN^Isolation^ (**k**) and MPN^Reunion^ (**l**) neurons on the basis of co-expression of activity induced *Fos* and neuron-type marker genes; *n* = 3–7 mice for each marker gene. All shaded areas and error bars represent the mean ± s.e.m. NS, not significant. All mouse brain diagrams were adapted from Paxinos and Franklin’s Mouse Brain Atlas^49^. Scale bars, 200 μm.

**Extended Data Fig. 2:**
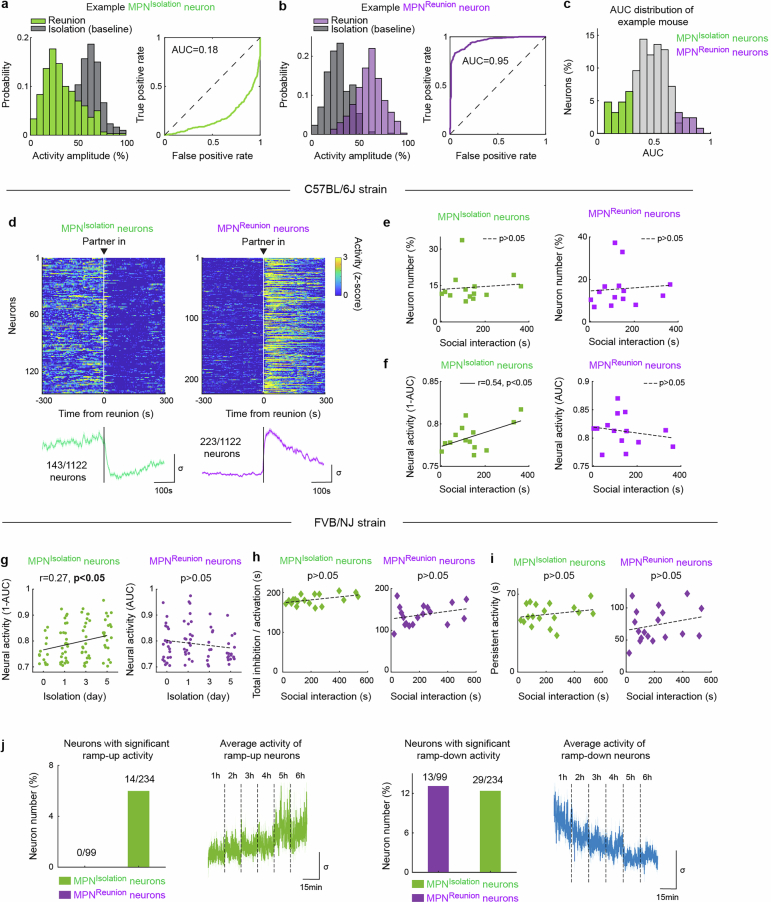
Identification of MPN neuron types responding to social isolation and social reunion using microendoscopy calcium imaging. **a-c**, Identification of MPN^Isolation^ and MPN^Reunion^ neurons using receiver operating characteristic (ROC) analysis. AUC, area under the ROC curve. **d**, Heatmap and average activity of MPN^Isolation^ or MPN^Reunion^ neurons in C57 mice after 3 days of isolation, n = 6 mice. **e**, **f**, Changes in number and neural activity of MPN^Isolation^ or MPN^Reunion^ neurons as a function of social rebound after various lengths of isolation in C57 mice, n = 4-6 mice. **g**, Neuronal activity changes after various durations of social isolation. Each dot represents a neuron, all neurons from an example FVB mouse. **h**, **i**, Analyses of total activation/inhibition durations (**h**) and persistent activity durations (**i**) during reunions. Each dot represents a mouse with average values across all neurons. n = 4-6 mice for different isolation durations. **j**, Analyses of MPN neurons with significant ramp-up and ramp-down activity during the first 6 h of isolation. Neuron numbers are noted above the bar graphs. n = 4 FVB mice. **e-i**, Pearson correlation analysis.

The observed rebound in social interaction following social deprivation indicates that homeostatic circuits, similar to those of physiological needs^12–17^, may exist in the hypothalamus to control social need and social satiety. To explore this hypothesis, we performed microendoscopy calcium imaging of pan-neuronal activity in the medial preoptic nucleus (MPN)—a hypothalamic nucleus involved in the control of social behaviours^27^—in freely interacting mice during social isolation and reunion (Fig. 2a,b). A receiver operating characteristic (ROC) analysis^28^ (Methods) was used to identify neuronal populations that are modulated significantly by social state (Extended Data Fig. 2a–c). Unique activity patterns were revealed in both FVB and C57 mice with non-overlapping populations activated during isolation and reunion (Fig. 2c, Extended Data Fig. 2d and Supplementary Video 2). Strikingly, neurons active during social isolation were inhibited promptly upon social reunion and re-activated upon removal of the social partner (Fig. 2c and Extended Data Fig. 2d). This population, referred to as MPN^Isolation^ neurons, tracked the isolation state in both FVB and C57 mice, and may thus signal social need. In both strains, we also observed another population, referred to as MPN^Reunion^ neurons, that stayed silent during isolation and became active during reunion (Fig. 2c and Extended Data Fig. 2d), thus potentially signalling social interaction. To examine the neural correlates of social need, we assessed the activity of MPN^Isolation^ and MPN^Reunion^ neurons as a function of the strength of social rebounds after isolation (Fig. 2d,e and Extended Data Fig. 2e,f). The number of MPN^Isolation^ neurons, identified on the basis of activity, increased and then plateaued with increased social rebound in FVB mice (Fig. 2d) but not C57 mice (Extended Data Fig. 2e), whereas the number of MPN^Reunion^ neurons remained stable in both strains (Fig. 2d and Extended Data Fig. 2e). Additionally, in both strains, the activity strength of MPN^Isolation^ neurons, but not MPN^Reunion^ neurons, correlated significantly with social rebound intensity (Fig. 2e and Extended Data Fig. 2f–i).

**Extended Data Fig. 3:**
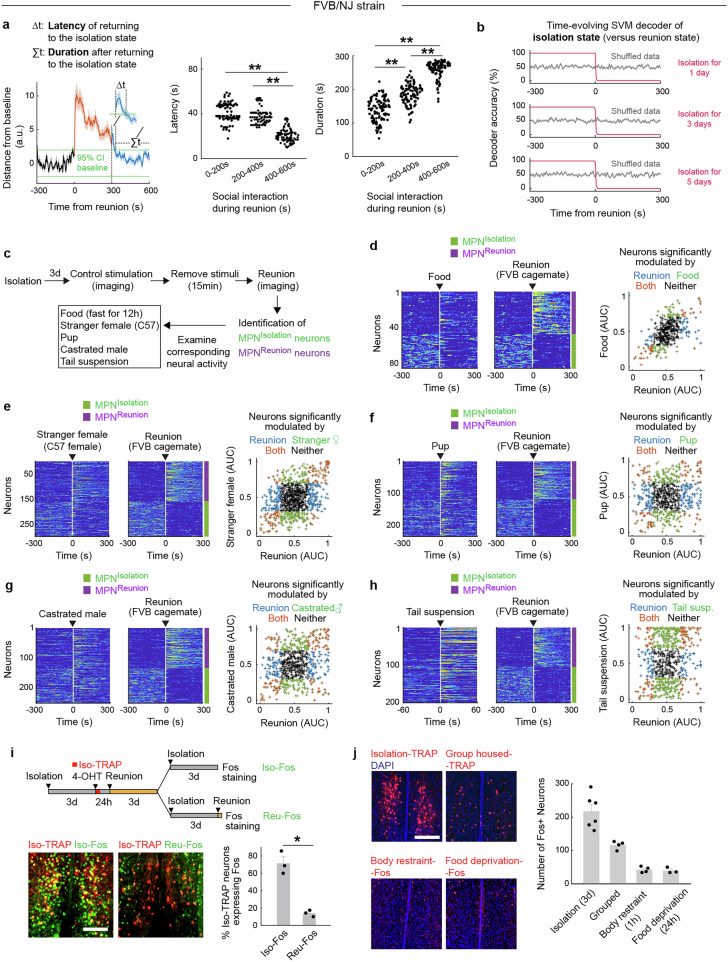
Assessment of MPN^Isolation^ and MPN^Reunion^ neuronal activity in different behavioral conditions. **a**, Analyses of population neural activity trajectory during re-isolation period after reunion. Each dot represents the result from a random subsampling of half imaged neurons. **p < 0.01, two-sample t-test. **b**, Decoding analyses of animal state switch using calcium imaging data and supporter vector classifiers. n = 4-6 mice. **c-h**, MPN neuronal activity during reunion and during other stimuli in FVB mice: food (**d**), a stranger female (C57 female, **e**), a pup (**f**), a castrated male (**g**), and tail suspension (**h**), n = 4 mice for all conditions. **i**, Cellular and spatial segregation of MPN isolation activated and reunion activated neurons. n = 3 mice, *p < 0.05, Mann–Whitney U test. **j**, Activity assessment of MPN neurons in FVB mice in distinct behavior contexts: social isolation, group housing, restraint stress (1 h) and food deprivation (24 h), n = 3-6 for each condition. Scale bars, 200 μm.

To gain insights into the emergence of social need, we monitored the activity of MPN^Isolation^ neurons in FVB mice at the onset of isolation for a period of 6h and uncovered sustained neuronal activity (Fig. 2f). The number of MPN^Isolation^ neurons increased in the first few hours of isolation (Fig. 2f) and a small population showed a significant ramp-up in activity as isolation progressed (Extended Data Fig. 2j). To examine the real-time neural representation of social state, we performed a principal component analysis (PCA) of population activity across MPN^Isolation^ and MPN^Reunion^ neurons during isolation and reunion. The PCA trajectory showed distinct neural representations for isolation and reunion states and a rapid state transition upon reunion (Fig. 2g,h). The trajectory did not return immediately to the initial isolation (baseline) state after removal of the social partner (Fig. 2g,h), but drifted back over minutes, with a faster return in the cohort of animals with high social rebound, indicating that social satiety was not reached during a brief reunion (Extended Data Fig. 3a). Support vector classifiers trained on 80% of MPN^Isolation^ and MPN^Reunion^ neuronal activity data robustly decode isolation versus reunion state in the held-out test data, indicating reliable neural representations of distinct social states (Extended Data Fig. 3b). Exposure to other behaviour contexts, such as food, stranger female, pup, castrated male and tail suspension, showed a high specificity in the activity of MPN^Isolation^ and MPN^Reunion^ neurons for social isolation and reunion, respectively (Fig. 2i and Extended Data Fig. 3c–h).

We also compared the activity patterns of MPN^Isolation^ and MPN^Reunion^ neurons in FVB and C57 mice and found that, after 3 days of isolation, the two strains exhibited similar numbers of MPN^Isolation^ and MPN^Reunion^ neurons (Fig. 2j). However, MPN^Isolation^ neurons showed stronger activity in FVB mice (Fig. 2j), indicating a more robust representation of social need.

## Molecular identity of activated neuronal populations

**Extended Data Fig. 4:**
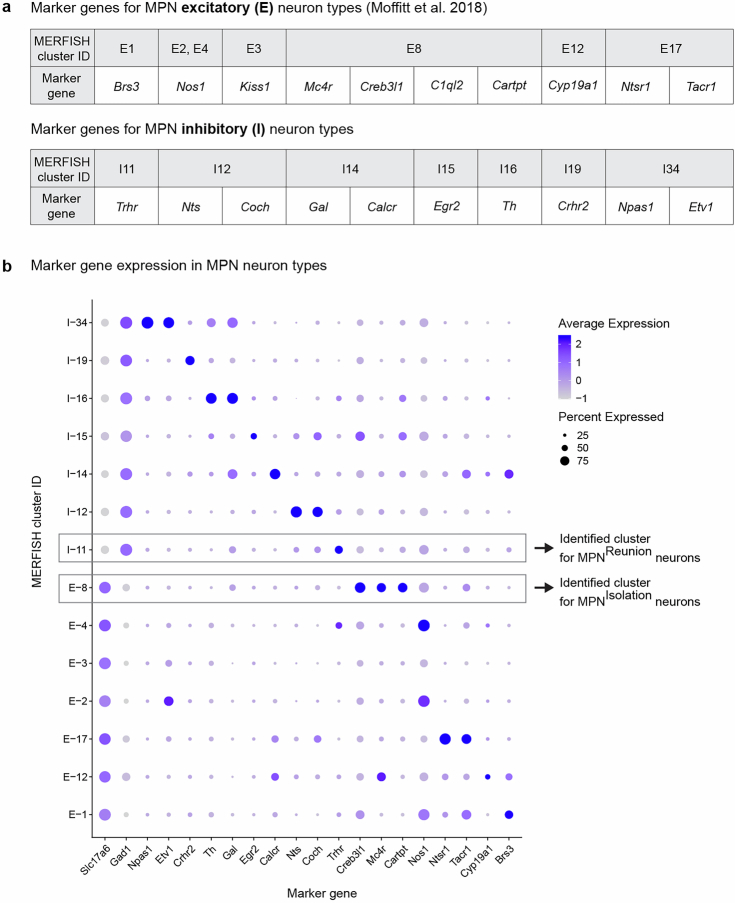
Marker genes for MPN neuronal cell types. **a**, Marker genes for different MPN excitatory and inhibitory neuronal populations based on Moffitt et al.^27^. **b**, Expression patterns of marker genes in different MPN clusters based on MERFISH experiments^27^. Boxes identify clusters that are best matched with MPN^Isolation^ and MPN^Reunion^ neurons.

**Extended Data Fig. 5:**
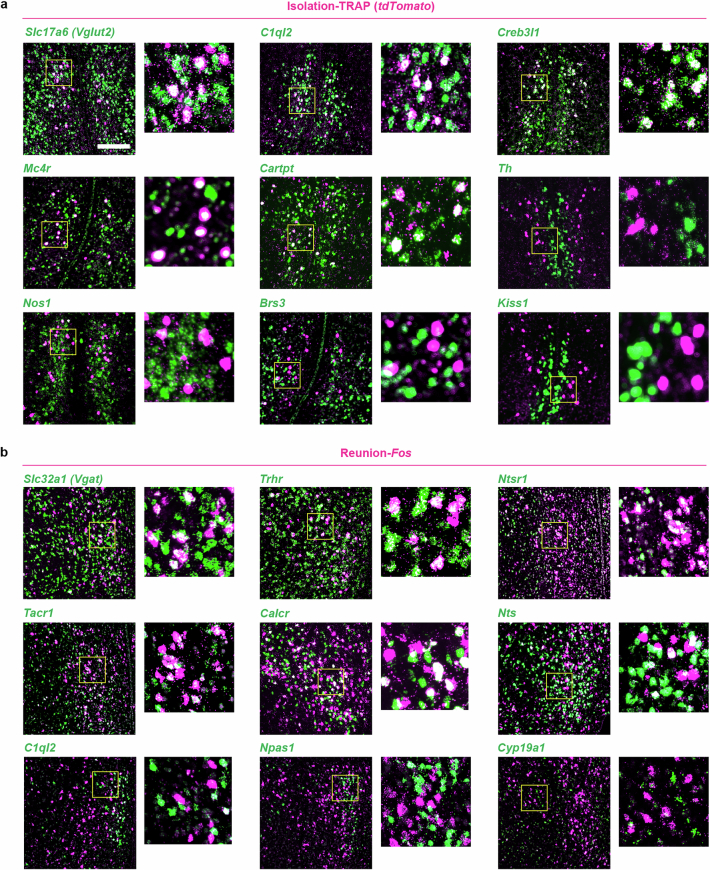
Cell-type identification of MPN^Isolation^ and MPN^Reunion^ neurons. **a, b**, Representative images of marker gene expression in distinct MPN cell-type clusters and overlap with MPN^Isolation^ (**a**) and MPN^Reunion^ (**b**) neurons using in situ hybridization. Green: marker genes for specific MPN neuron types, magenta: *Fos* expression during social isolation (**a**) or social reunion (**b**). Scale bar, 200 μm (all images). Zoom-in boxes, 150 μm × 150 μm.

To identify candidate hypothalamic populations underlying the control of social need and social satiety, we used the activity-dependent and tamoxifen-inducible Cre line TRAP2 (ref. ^16^), crossed to a reporter line Ai9, to label activated neurons across hours during social isolation (Fig. 2k) and *Fos* in situ hybridization to visualize transiently activated neurons during social rebound in the FVB strain (Fig. 2l). We confirmed that MPN neurons activated by isolation and reunion represent two distinct populations (Extended Data Fig. 3i) and are not activated by group housing or generic stress such as body restraint or food deprivation (Extended Data Fig. 3j). We sought to assign these populations to our recently established spatial transcriptomic cell-type atlas in the preoptic region^27^. Co-expression of *Fos* and cell-type marker genes (Extended Data Fig. 4) showed that MPN^Isolation^ neurons are glutamatergic (*Slc17a6*^+^) and enriched in *Mc4r*, *Cartpt*, *Creb3l1* and *C1ql2*, which matches MERFISH cluster E8 (ref. ^27^) (Fig. 2k and Extended Data Figs. 4 and 5a), whereas most (around 80%) of MPN^Reunion^ neurons are GABAergic (*Slc32a1*^+^) and enriched in *Trhr*, corresponding to MERFISH cluster I11 (Fig. 2l and Extended Data Figs. 4 and 5b).

**Extended Data Fig. 6:**
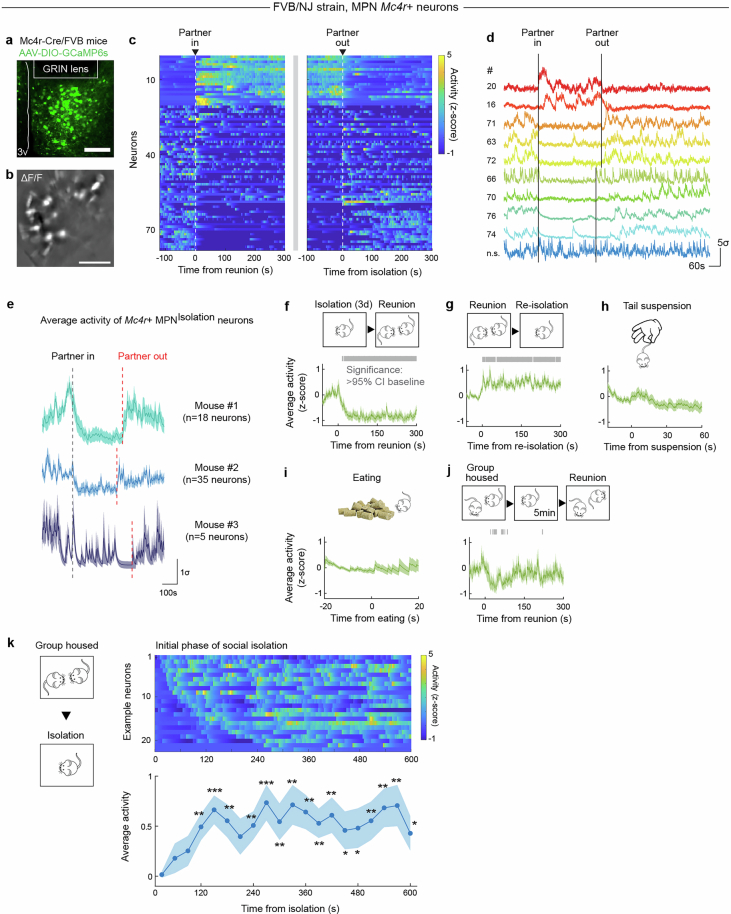
Microendoscopy calcium imaging of MPN^*Mc4r*+^ neurons. **a**, Representative image showing MPN^*Mc4r*+^ neurons expressing GCaMP6s. **b**, Example imaging field of GRIN lens, represented as ΔF/F. **c**, MPN^*Mc4r*+^ neuronal activity during social isolation and social reunion. n = 78 significantly modulated neurons pooled from 3 mice. **d**, Calcium activity of 10 example imaged MPN^*Mc4r*+^ neurons. The top 9 neurons were selected from (**c**) as significantly modulated, while the bottom neuron showed non-significant modulation (n.s.). **e**, Average calcium activity of MPN^*Mc4r*+^ neurons that were inhibited during reunion, n = 3 mice. **f-j**, Average calcium activity of reunion-inhibited MPN^*Mc4r*+^ neurons in distinct behavioral regimes. n = 3 mice. Grey bars indicate the significance of activity above or below 95% confidence interval of baseline. **k**, Calcium imaging of reunion-inhibited MPN^*Mc4r+*^ neurons in the initial phase of social isolation. The trace below represents the average activity across 27 neurons pooled from 2 mice. Wilcoxon signed-rank tests were used to estimate statistical significance of enhanced neuronal activity in each time bin (30 s) versus the first bin, *p < 0.05, **p < 0.01, ***p < 0.001. All shaded areas and error bars represent the mean ± s.e.m.

Next, we assessed the activity of MPN^*Mc4r*^ neurons in FVB mice using microendoscopy calcium imaging (Extended Data Fig. 6a,b). The majority of the imaged MPN^*Mc4r*^ neurons were activated by social isolation and inhibited upon social reunion (67 ± 8% of responsive neurons, a threefold enrichment compared with pan-neuronal imaging), but not by other control behaviours (Extended Data Fig. 6c–i), thus confirming their identity as the MPN^Isolation^ population. MPN^Isolation^ neurons were transiently inhibited in group-housed mice reunited after a 5-min isolation (Extended Data Fig. 6j) and exhibited increased activity at the onset of isolation (Extended Data Fig. 6k).

## Functional characterization of MPN^Isolation^ neurons

**Fig. 3:**
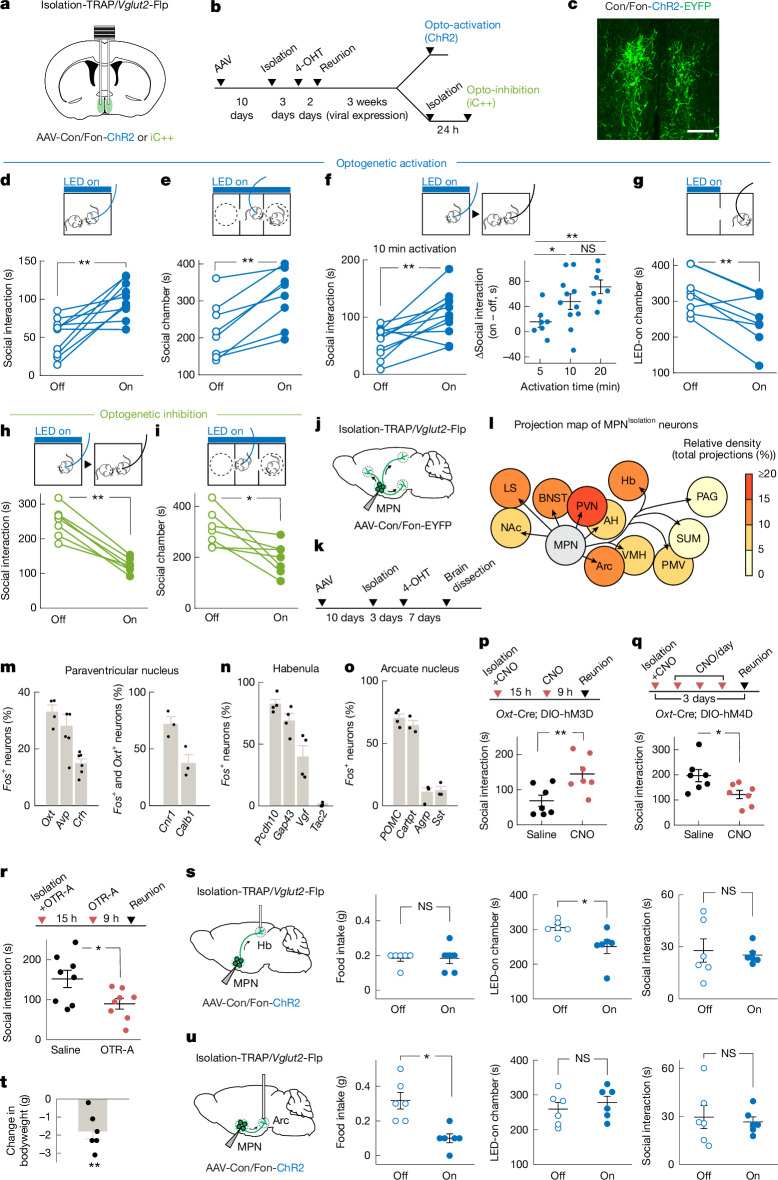
Functional characterization of MPN^Isolation^ neurons. **a**,**b**, Intersectional (Cre/Flp) strategy (**a**) to target MPN^Isolation^ neurons for optogenetic manipulations (**b**). **c**, Representative image of ChR2-expressing MPN^Isolation^ neurons. **d**–**g**, Behavioural effects of optogenetic activation of MPN^Isolation^ neurons in group-housed mice: activation during social interaction, *n* = 10 (**d**); three-chamber social preference test, *n* = 8 (**e**); pre-interaction stimulation, n = 7–11 (**f**) and real-time conditioned place preference test, *n* = 8 (**g**). **h**,**i**, Optogenetic inhibition of MPN^Isolation^ neurons before social reunion, *n* = 8 (**h**) and during three-chamber social preference test, *n* = 7 (**i**). **j**,**k**, Viral tracing strategy (**j**) to map projections from MPN^Isolation^ neurons (**k**). **l**, Projection map of MPN^Isolation^ neurons, *n* = 4 mice. AH, anterior hypothalamus; Hb, habenula; LS, lateral septum; PAG, periaqueductal grey. **m**–**o**, Identification of the activated neurons in projection regions (PVN (**m**), habenula (**n**) and Arc (**o**)) of MPN^Isolation^ neurons; *n* = 3–6 mice. **p**,**q**, Behavioural effects of chemogenetic activation (**p**) and inhibition (**q**) of oxytocin neurons in the PVN during social isolation; *n* = 7 mice. **r**, Effect of oxytocin receptor antagonist (OTR-A, intraperitoneal) during isolation on subsequent reunion; *n* = 8 mice. **s**,**u**, Behavioural effects of optogenetic activation of MPN^Isolation^ towards habenula (**s**) and MPN^Isolation^ towards Arc (**u**) projections on eating, real-time place preference and social behaviours; *n* = 6 mice. **t**, Changes in mouse bodyweight during isolation compared with group-housed condition; *n* = 6 mice. Wilcoxon signed-rank tests (**d**–**i**, **s**–**u**); Mann–Whitney *U-*test (**p**–**r**); *P < 0.05, **P < 0.01. NS, not significant. All error bars represent the mean ± s.e.m. All mouse brain diagrams were adapted from Paxinos and Franklin’s Mouse Brain Atlas^49^. Scale bar, 200 μm (**c**).

**Extended Data Fig. 7:**
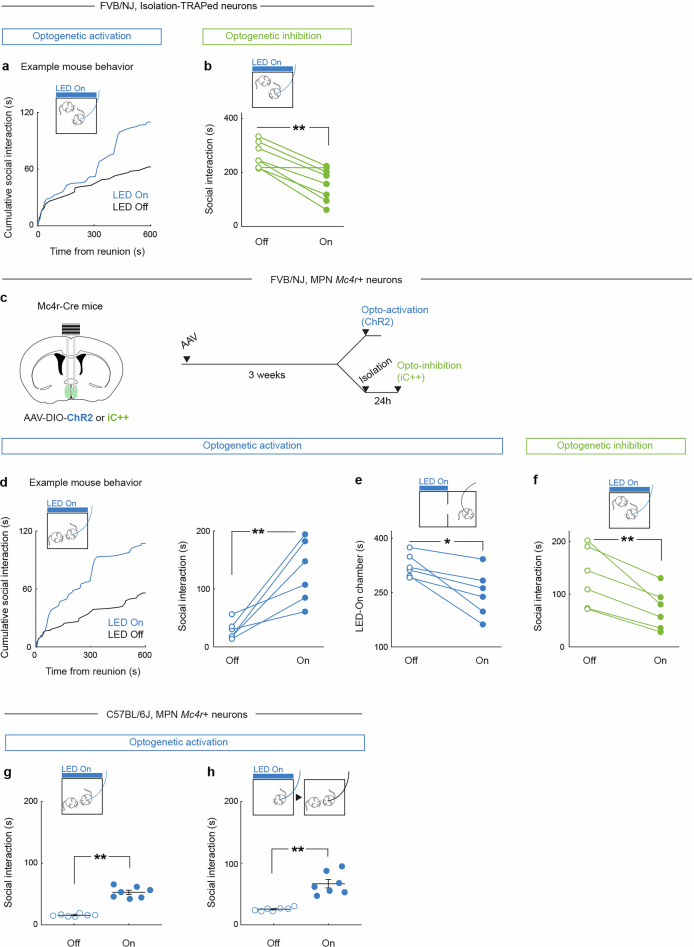
Optogenetic manipulations of MPN^Isolation^ neurons. **a**, Behavioral effect of optogenetic activation of MPN^Isolation^ neurons during social interaction. Curves showing cumulative behavior from an example mouse with or without LED illumination. **b**, Behavioral effect when inhibiting MPN^Isolation^ neurons during social reunion. n = 8. **c**, Strategy to target MPN^*Mc4r*+^ neurons for optogenetic manipulations. **d**, **e**, Behavioral effects of optogenetic activation of MPN^*Mc4r*+^ neurons during social interaction (**d**) or real-time place preference test (**e**) in FVB mice, n = 6 for both conditions. **f**, Behavioral effect of optogenetic inhibition of MPN^Mc4r+^ neurons during social reunion in FVB mice, n = 6. **g**, **h**, Behavioral effects of optogenetic activation of MPN^*Mc4r*+^ neurons in C57 mice during or before social interaction, n = 7. **b**, **d-h**, Wilcoxon signed-rank tests; *p < 0.05, **p < 0.01. The mouse brain diagram in panel **c** was adapted from Paxinos and Franklin’s Mouse Brain Atlas^49^.

We used optogenetics to examine the functional contribution of MPN^Isolation^ neurons to the emergence of social need. To specifically target MPN^Isolation^ neurons, we expressed Cre- and Flp-dependent ChR2 (Con/Fon-ChR2) in TRAP2/*Vglut2*-Flp mice in which the expression of Cre recombinase is induced by injection of 4-hydroxytamoxifen (4-OHT) during social isolation (Fig. 3a–c). Optogenetic activation of MPN^Isolation^ neurons in group-housed FVB mice, whose social need is satiated, induced significant social interaction (Fig. 3d and Extended Data Fig. 7a) and enhanced social preference in a three-chamber sociability assay (Fig. 3e). Notably, optogenetic activation applied before reunion also led to a subsequent rebound in social contact that scaled with the duration of activation (Fig. 3f), indicating a sustained, dose-dependent effect of a transient activation of MPN^Isolation^ neurons. Place preference assay showed that mice avoided the chamber coupled with optogenetic activation (Fig. 3g), indicating that MPN^Isolation^ neuronal activity conveys negative valence, and thus may mediate the aversive emotional state associated with isolation. Similarly, we used an intersectional approach to inhibit MPN^Isolation^ neurons with the light-activated chloride channel iC++ and observed a reduced social rebound when the inhibition was applied either before (Fig. 3h) or during (Extended Data Fig. 7b) reunion. MPN^Isolation^ neuronal inactivation also significantly reduced social preference after isolation (Fig. 3i). Similar results were obtained by optogenetic manipulations of MPN^*Mc4r*^ neurons in both FVB and C57 mice (Extended Data Fig. 7c–h), indicating conserved function across mouse strains.

Altogether, the activity of MPN^Isolation^ neurons seems to be essential to the expression of social need and is sufficient to trigger social interaction in socially satiated mice.

## Neural circuits of MPN^Isolation^ neurons

**Extended Data Fig. 8:**
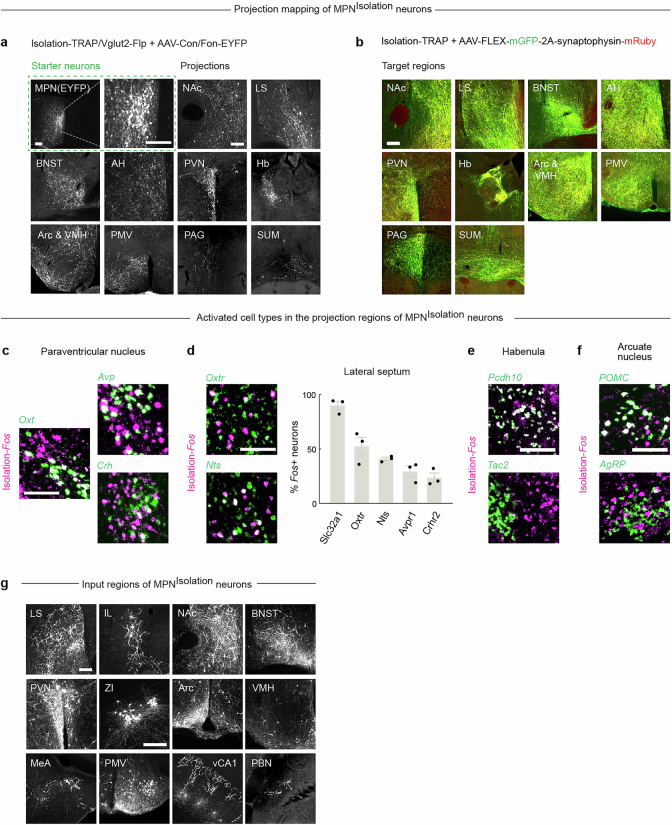
Circuit tracing from MPN^Isolation^ neurons. **a**-**b**, Representative images showing downstream targets of MPN^Isolation^ neurons using intersectional (Cre/Flp, **a**) and TRAP (**b**) methods, n = 2-3. All scale bars, 200 μm. **c-f**, Cell type identification of the activated neurons in the projection regions of MPN^Isolation^ neurons. Green: marker genes, magenta: *Fos* expression following social isolation. All scale bars, 100 μm. **g**, Representative images of input regions of MPN^Isolation^ neurons. Scale bars, 200 μm. Images without scale bars have the same scale as the first image in each panel.

Social isolation leads to an aversive emotional state^21^ (Fig. 3g) and modulates a wide range of behaviours, such as increased social seeking and altered appetite^5,6^. To investigate how MPN^Isolation^ neurons exert these functions, we identified their downstream targets by viral expression of enhanced yellow fluorescent protein (EYFP) (Fig. 3j–l and Extended Data Fig. 8a). Projections of MPN^Isolation^ neurons were found mostly in hypothalamus (paraventricular nucleus of hypothalamus (PVN), Arc, ventromedial hypothalamus (VMH), supramammillary nucleus (SUM), ventral premammillary nucleus (PMV)), in regions conveying aversive emotions (lateral septum, bed nucleus of the stria terminalis (BNST), habenula) and in downstream motor relay (periaqueductal grey) (Fig. 3l and Extended Data Fig. 8a). These results were confirmed with a conditional virus expressing synaptophysin–mRuby and membrane-bound green fluorescent protein (GFP) in MPN^Isolation^ neurons to delineate synapses from fibres of passage (Extended Data Fig. 8b). Next, we characterized the identity and function of activated neurons in four principal downstream regions showing strong *Fos* activation during isolation: PVN, habenula, Arc and lateral septum. Social isolation led to the activation of *Oxt*^+^, *Avp*^+^ neurons in PVN and *Oxtr*^+^ neurons in lateral septum (Fig. 3m and Extended Data Fig. 8c,d), indicating that the oxytocin system may underlie the enhanced social drive triggered by isolation. Indeed, chemogenetic activation of PVN^*Oxt*^ neurons during isolation enhanced social rebound during reunion, whereas inhibition led to a reduced social rebound compared with a control group (Fig. 3p,q). Moreover, intraperitoneal injection of oxytocin receptor antagonist (OTR-A) during isolation significantly reduced social rebound (Fig. 3r). Next, we found that *Gap43*^+^, *Pcdh10*^+^ and *Vgf*^+^ neurons in the lateral habenula (LHb)^29,30^ (Fig. 3n and Extended Data Fig. 8e), *Nts*^+^ and *Crhr2*^+^ neurons in lateral septum^31,32^ (Extended Data Fig. 8d) and *Crh*^+^ neurons in PVN (Fig. 3m), all previously shown to process negative emotions, were activated during social isolation. Indeed, expression of ChR2 in MPN^Isolation^ neurons and stimulation of projections in LHb induced real-time avoidance but did not affect eating or social behaviours (Fig. 3s). *POMC*^+^ and *Cartpt*^+^ appetite-suppressing neurons in Arc were also activated during social isolation (Fig. 3o and Extended Data Fig. 8f), consistent with the observed bodyweight loss (Fig. 3t). Optogenetic activation of MPN^Isolation^-to-Arc projections inhibited food intake but did not induce negative valence or affect social interaction (Fig. 3u), indicating inhibition of hunger by social need. The distinct behavioural effects elicited by stimulations of MPN^Isolation^-to-LHb and MPN^Isolation^-to-Arc projections rule out possible cross-talk between these projections through backpropagated action potentials induced by terminal activation. Together, these results showed discrete neural circuits downstream of MPN^Isolation^ neurons that mediate distinct aspects of isolation state.

## MPN^Reunion^ neurons modulate social satiety

**Fig. 4:**
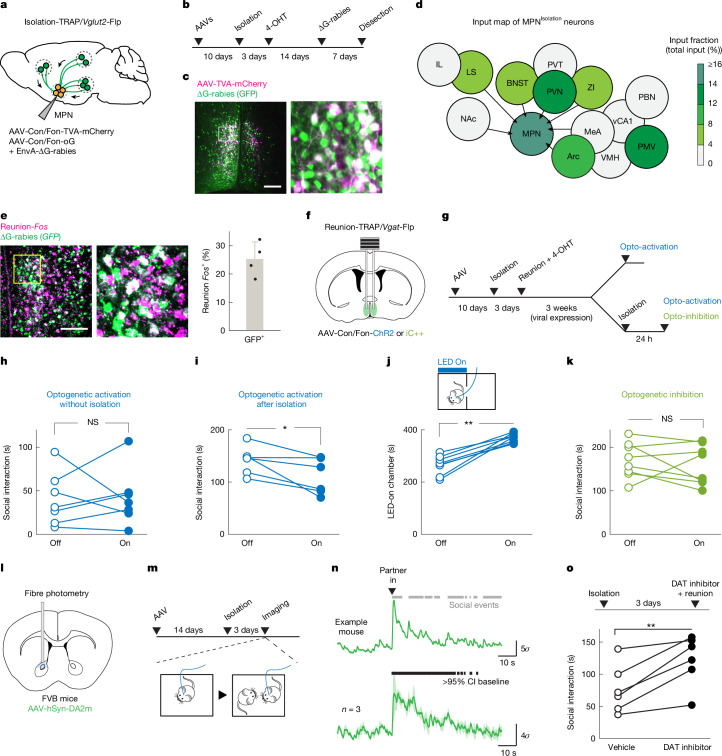
MPN^Reunion^ neurons modulate social satiety. **a**,**b**, Intersectional (Cre/Flp) viral tracing strategy (**a**) to map input brain regions of MPN^Isolation^ neurons (**b**). **c**,**d**, Representative image of MPN^isolation^ starter neurons (**c**) and input brain regions; *n* = 5 mice (**d**). IL, infralimbic cortex; MeA, medial amygdala; vCA1, ventral hippocampal CA1; ZI, zona incerta. **e**, Representative image and quantification of MPN^Reunion^ neurons projecting to MPN^Isolation^ neurons; *n* = 4 mice. **f**,**g**, Intersectional (Cre/Flp) strategy (**f**) to target MPN^Reunion^ neurons for optogenetic manipulations (**g**). **h**–**j**, Behavioural effects of optogenetic activation of MPN^Reunion^ neurons in different conditions: without isolation, *n* = 7 (**h**); after isolation, *n* = 6 (**i**) and real-time place preference test, *n* = 7 (**j**). **k**, Behavioural effect of optogenetic inhibition of MPN^Reunion^ neurons during social reunion, n = 8. **l**,**m**, Experimental strategy to image dopamine release in the NAc (**l**) during social reunion (**m**). **n**, Dopamine release in the NAc upon social reunion in FVB mice. Grey bars (upper) indicate social events and black bars (lower) indicate the significance of enhanced activity above 95% confidence interval of the baseline; *n* = 3 mice. **o**, Behavioural effect of dopamine transporter inhibitor infusion into the NAc during social reunion; *n* = 6. Wilcoxon signed-rank tests (**h**–**k**, **o**); *P < 0.05, **P < 0.01. NS, not significant. Shaded areas and all error bars represent the mean ± s.e.m. All mouse brain diagrams were adapted from Paxinos and Franklin’s Mouse Brain Atlas^49^. Scale bars, 200 μm (**c**, **e**).

To understand circuit mechanisms underlying the inhibition of MPN^Isolation^ neurons upon social reunion, we performed monosynaptic retrograde rabies tracing^33^ from MPN^Isolation^ neurons (Fig. 4a,b) and identified 12 input areas (Fig. 4c,d and Extended Data Fig. 8g), many overlapping with MPN^Isolation^ projections (Fig. 3l), thus showing extensive recurrent connectivity within the isolation circuit. We noticed strong input signals to MPN^Isolation^ neurons in the preoptic area and identified MPN^Reunion^ neurons as directly synapsing onto MPN^Isolation^ neurons on the basis of co-expression of presynaptic rabies (expressing GFP) from MPN^Isolation^ neurons and *Fos* induced by social reunion (Fig. 4e). Because around 80% of MPN^Reunion^ neurons are GABAergic, their activation may directly inhibit MPN^Isolation^ neurons during social rebound, thus serving as a social satiety signal.

**Extended Data Fig. 9:**
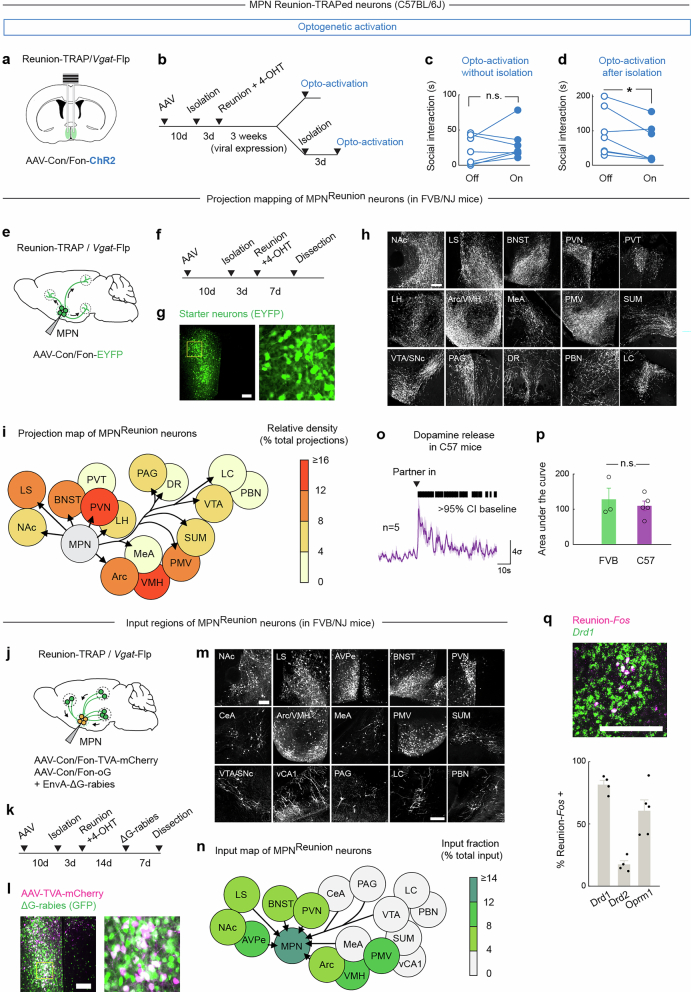
Circuit tracing from MPN^Reunion^ neurons. **a-d**, Experimental strategy and behavioral effects of optogenetic activation of MPN^Reunion^ neurons in C57 mice, n = 7. *p < 0.05, Wilcoxon signed-rank test. **e**,**f**, Viral tracing strategy to map target brain regions of MPN^Reunion^ neurons. **g**, Representative images of MPN^Reunion^ starter neurons. **h**, Representative images of projections from MPN^Reunion^ neurons. **i**, Schematic summary of projections from MPN^Reunion^ neurons and relative projection density, n = 4 mice. **j**,**k**, Viral tracing strategy to map input brain regions of MPN^Reunion^ neurons. **l**, Representative images of MPN^Reunion^ starter neurons in retrograde tracing. **m**, Representative images of input regions of MPN^Reunion^ neurons. **n**, Schematic summary of input brain regions of MPN^Reunion^ neurons and their relative input intensity, n = 4 mice. **o**, Dopamine release in the NAc upon social reunion in C57 mice, n = 5. **p**, Comparison of dopamine release in the FVB and C57 strains. n = 3-5 mice. n.s., not significant, Mann–Whitney U test. **q**, Cell type identification of NAc neurons that are activated during social reunion. n = 4-5 mice. All scale bars, 200 μm. Images without scale bars in **h** and **p** have the same scale as in the first images. Abbreviations: MPN, medial preoptic nucleus. NAc, nucleus accumbens. LS, lateral septum. AVPe, anteroventral periventricular nucleus. BNST, bed nucleus of the stria terminalis. PVN, paraventricular nucleus of hypothalamus. PVT, paraventricular thalamus. LH, lateral hypothalamus. CeA, central amygdala. Arc, arcuate nucleus. VMH, ventromedial hypothalamus. MeA, medial amygdala. PMV, ventral premammillary nucleus. SUM, supramammillary nucleus. VTA, ventral tegmental area. SNc, substantia nigra pars compacta. vCA1, ventral hippocampal CA1. PAG, periaqueductal gray. DR, dorsal raphe nucleus. PBN, parabrachial nucleus. LC, locus coeruleus. All mouse brain diagrams were adapted from Paxinos and Franklin’s Mouse Brain Atlas^49^.

To test this hypothesis, we targeted MPN^Reunion^ neurons by expressing Con/Fon-ChR2 in TRAP2/*Vgat*-Flp FVB mice and inducing the expression of Cre by injection of 4-OHT during social reunion (Fig. 4f,g). Optogenetic activation of MPN^Reunion^ neurons did not affect social interaction in group-housed animals (Fig. 4h) but led to a decreased social rebound in isolated mice (Fig. 4i), supporting a role for MPN^Reunion^ neurons in mediating social satiation. A similar social satiation effect was observed when MPN^Reunion^ neurons were activated in the C57 strain (Extended Data Fig. 9a–d). Mice preferred to stay in the chamber associated with stimulation of MPN^Reunion^ neurons, indicating a positive valence conveyed by their activity (Fig. 4j). Inhibition of MPN^Reunion^ neurons during reunion did not increase the rebound (Fig. 4k), perhaps due to the incomplete capture of MPN^Reunion^ neurons by the TRAP approach or the existence of other social satiation mechanisms.

To gain a broader understanding of the neural circuitry underlying social homeostasis, we further mapped the input and output of MPN^Reunion^ neurons and identified several brain regions, such as lateral septum, NAc, BNST, PVN and Arc, also associated with MPN^Isolation^ neuronal circuits (Extended Data Fig. 9e–n), indicating shared neural networks regulating social need and social satiety. MPN^Reunion^ neurons showed dense projections to the ventral tegmental area (VTA), known to have robust dopaminergic projections to the nucleus accumbens (NAc) associated with social reward^19^. We expressed the dopamine sensor GRAB_DA2m_^34^ in NAc, measured fluorescence changes with fibre photometry and observed significant dopamine release upon social reunion in both FVB and C57 mice (Fig. 4l–n and Extended Data Fig. 9o,p). To examine the contribution of dopamine release to social rebound, we infused a dopamine transporter inhibitor using bilateral cannulas in NAc right before reunion to block the reuptake of dopamine, thus boosting dopamine signalling. A rise in dopamine signalling resulted in a significant increase in social rebound (Fig. 4o), indicating an enhanced social motivation mediated by dopamine release. We found that NAc neurons activated by reunion express the dopamine D1 receptor and the opioid receptor *Oprm1* (Extended Data Fig. 9q), consistent with the neuronal populations associated with social reward^35^.

These results identify a brain-wide neural circuitry underlying social homeostasis. Social stimuli activate MPN^Reunion^ neurons, which further recruit downstream social reward circuits and inhibit MPN^Isolation^ neurons. Conversely, during social isolation, absence of sensory input silences MPN^Reunion^ neurons and in turn dis-inhibits MPN^Isolation^ neurons, which further triggers negative valence, social motivation and other physiological changes associated with social isolation.

## Sensory basis of social homeostasis

**Fig. 5:**
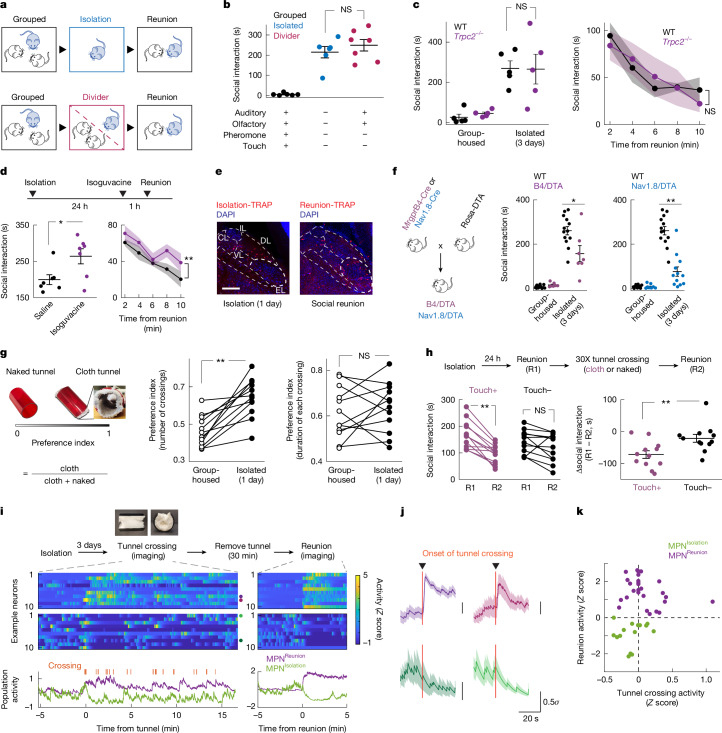
Sensory basis of social need and social satiety. **a**,**b**, Assessment of sensory contribution to social rebound (**a**); *n* = 6 for standard isolation, *n* = 7 for divider experiments. Plus and minus symbols indicate the presence and the absence of a given sensory modality, respectively (**b**). **c**, Social rebound and satiation in *Trpc2*^−/−^ mice; *n* = 5. **d**. Behavioural effect of acute inhibition of touch sensation (intraperitoneal injection of isoguvacine) during social reunion; *n* = 7. **e**, Representative images of neuronal activity in PBN subnuclei (CL, central lateral; DL, dorsal lateral; EL, external lateral; IL, Internal lateral; VL, ventral lateral) during social isolation and social reunion. **f**, Effects of genetic ablation of Mrgprb4-lineage neurons or Nav1.8^+^ neurons on social rebound; *n* = 7–13 for each condition. **g**, Touch preference assay in group-housed and isolated mice; *n* = 12. **h**, Effect of cloth or naked tunnel crossing on social rebound; *n* = 12. **i**, Gentle touch modulates the activity of MPN^Reunion^ and MPN^Isolation^ neurons. The activity of ten representative neurons was plotted in the heatmap. Green and purple curves represent the average activity of all identified MPN^Isolation^ (*n* = 15) and MPN^Reunion^ (*n* = 27) neurons, respectively. **j**, Example neurons that were activated (*n* = 2, upper) or inhibited (*n* = 2, lower) by cloth tunnel crossing. **k**, Neuronal activity (z-scores subtracting baseline) during social reunion versus cloth tunnel crossing. Dots represent single neurons. *n* = 15 MPN^Isolation^ neurons, *n* = 27 MPN^Reunion^ neurons. **b**, Kruskal–Wallis test. Mann–Whitney *U-*test: **c**, left; **d**, left; **f**,**h**, right. Two-way ANOVA: **c**, right; **d**, right. Wilcoxon signed-rank tests: **g**,**h**, left. NS, not significant; *P < 0.05, **P < 0.01. All shaded areas and error bars represent the mean ± s.e.m. Scale bar, 200 μm (**e**).

**Extended Data Fig. 10:**
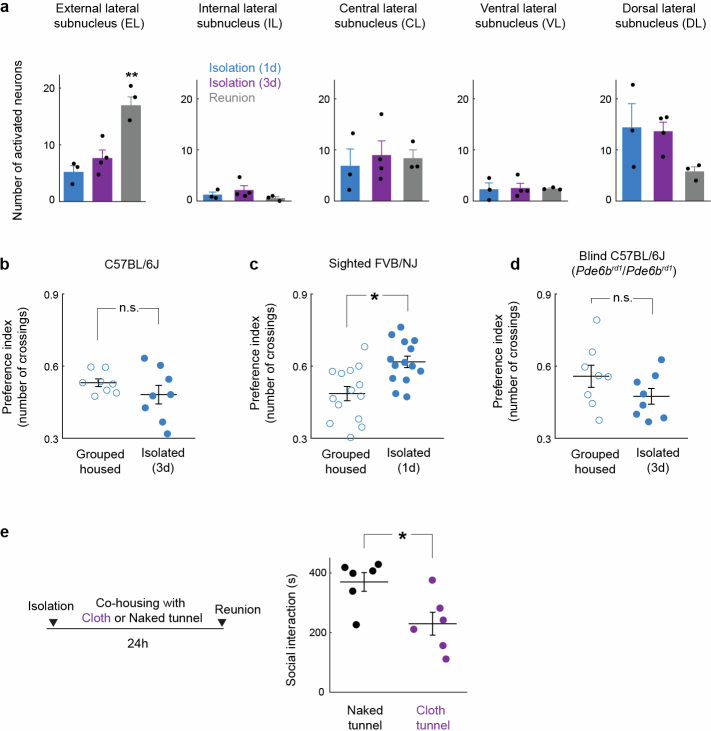
Modulation of social need by touch. **a**, Quantification of activated neurons in PBN subnuclei during social isolation and reunion, n = 3-4 in each condition. Kruskal-Wallis test. **b-d**, Touch preference test in C57BL/6 J mice, n = 8 (**b**), sighted FVB mice, n = 14 (**c**), and blind C57 mice, n = 8 (**d**). Preference index is the ratio of crossing numbers through cloth tunnel over total crossings. *p < 0.05, Wilcoxon signed-rank tests; n.s., not significant. **e**, Behavioral effect of co-housing with cloth or naked tunnel during isolation on social interaction during reunion, n = 6. Mann–Whitney U test, *p < 0.05. All error bars represent the mean ± s.e.m.

How do animals sense whether they are alone or together? Sighted and blind FVB mice showed similar amounts of social rebound (Extended Data Fig. 1o–q), indicating that visual cues do not contribute significantly to social homeostasis. To further investigate contributions of various senses, FVB mice were separated from siblings in their home cage by a perforated divider, such that auditory and olfactory cues could be sensed by animals across the divider (Fig. 5a). Surprisingly, a significant social rebound, comparable with that induced in singly housed mice, was observed after 3 days of separation (Fig. 5b), indicating that signals crossing the divider—auditory and olfactory cues—are not sufficient to fulfil social need. Next, we tested two sensory modalities that require direct contact—pheromone sensing and touch. *Trpc2*^−/−^ mice, which are genetically impaired in vomeronasal pheromone sensing^36^, showed normal social rebound after isolation and normal satiation during reunion (Fig. 5c). By contrast, mice treated before social reunion with intraperitoneal injection of isoguvacine, a peripherally restricted GABA_A_ receptor agonist that attenuates activity in several mechanosensory neuron types^37^, showed prolonged social rebound (Fig. 5d), indicating a delay in the satiation of social need. Social rebound robustly activates the external lateral subnucleus of the parabrachial nucleus (PBN_EL_) (Fig. 5e and Extended Data Fig. 10a)—a region conveying touch information from the spinal cord to the brain and implicated in affective touch^38^. Next, we crossed *Mrgprb4*-Cre (B4-Cre) and *Nav1.8*-Cre mice to a Cre-dependent line expressing diphtheria toxin subunit A (DTA) to ablate neuron types in the dorsal root ganglia implicated in social touch^39–41^. We found that mice from both crosses (B4/DTA and Nav1.8/DTA) showed a significant reduction in social rebound compared with wild-type controls (Fig. 5f), indicating that the constitutive loss of one or more mechanosensory neuronal populations reduces sensitivity to social environment and hampers the generation of social drive during isolation. The reduced but still significant rebound (Fig. 5f) indicates that other types of mechanosensory neurons, presumably A-fibre low threshold mechanoreceptors, which are spared in Nav1.8/DTA mice, also contribute to the sensation of social context. Collectively, these results support a model in which several mechanosensory neuron types contribute to social need regulation.

Inspired by Harlow’s pioneering work^42^, in which separated infant rhesus monkeys strongly preferred, and attached to, a soft cloth surrogate mother rather than a rigid wire mother, we designed a comfort-touch preference assay adapted to mice. Mice were exposed to two tunnels, one internally coated with soft cloth (cloth tunnel) and the other left naked (naked tunnel) (Fig. 5g and Supplementary Video 3). After 1 day of isolation, but not when group-housed, FVB mice showed a significant preference for crossing the cloth versus naked tunnel as quantified by the relative number of crossings (Fig. 5g, left graph). The relative duration of crossings was similar between group and isolated conditions (Fig. 5g, right graph), indicating that the preference of the cloth tunnel results from touch stimulation rather than shelter or warmth seeking. C57 mice, which show low social rebound, did not show such preference even after longer isolation (Extended Data Fig. 10b). We observed a similar touch preference in sighted FVB, but not blind C57 mice (Extended Data Fig. 10c,d), ruling out the contribution of blindness to touch preference. To investigate whether comfort-touch can help fulfil social need, we let isolated mice cross either cloth or naked tunnels 30 times and measured their social rebound before and after crossing. Notably, soft-touch-stimulated mice showed significantly lower rebound after crossing, whereas mice running through naked tunnels showed a similar rebound as before (Fig. 5h). Similarly, co-housing during isolation with a cloth, but not naked, tunnel reduced social rebound (Extended Data Fig. 10e).

Does soft touch affect the activity of MPN^Reunion^ and MPN^Isolation^ neurons? We modified the cloth tunnel with a top opening to allow mice to run through with a head implant and attached wire for microendoscopy calcium imaging. After 3 days of isolation, implanted mice were allowed to freely access cloth tunnels for 15 min (10–20 crossings) while imaging MPN neuronal activity. After removal of tunnels, a 5-min reunion was applied to identify MPN^Reunion^ and MPN^Isolation^ neurons on the basis of their activity patterns and the activity of these two populations during previous cloth tunnel crossings was examined (Fig. 5i). Strikingly, soft touch inhibited more than 90% of MPN^Isolation^ neurons and excited roughly 35% of MPN^Reunion^ neurons (Fig. 5i–k), thus mimicking signals underlying social satiation. Overall, these results indicate that touch is a key sensory modality for mice to perceive social environment, with lack of touch sensation leading to the emergence of social need, and its presence providing social satiety.

## Discussion

**Fig. 6:**
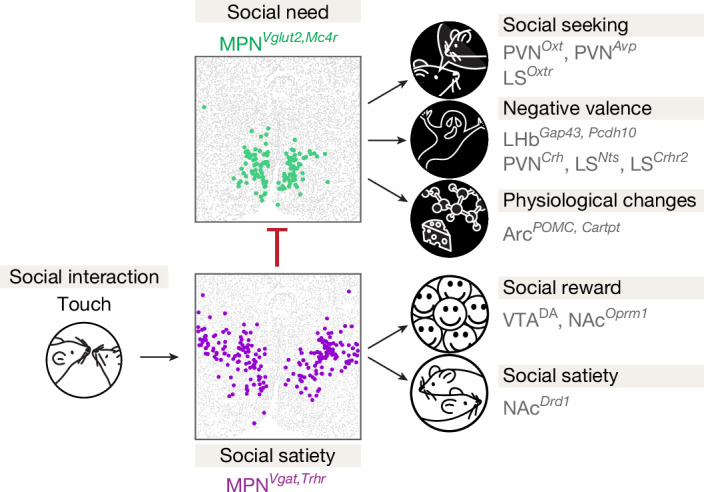
Model of neural circuits underlying social homeostasis. Gentle touch associated with social interaction leads to activation of MPN^Reunion^ neurons, recruitment of social reward circuits, inhibition of MPN^Isolation^ neurons and social satiety. Conversely, absence of social touch during isolation inactivates MPN^Reunion^ neurons and in turn dis-inhibits (activates) MPN^Isolation^ neurons, which induces social drive, negative valence and modulation of other physiological functions that constitute the isolation state.

In this study, we characterized two interconnected hypothalamic neuronal populations that form a key regulatory node underlying social homeostasis (Fig. 6). MPN^Isolation^ neurons (MPN^*Vglut2*, *Mc4r*, *Cartpt*^) are active during social isolation and silent upon social reunion. They convey negative valence and promote, and are required for, social interaction triggered by social isolation, indicating an essential role in encoding social need when animals are socially deprived. By contrast, MPN^Reunion^ neurons (MPN^*Vgat,Trhr*^) are active during social reunion and silent during social isolation. Their activation reduces social interaction and conveys positive valence, indicating a role in social satiation during reunion after isolation. MPN^isolation^ and MPN^Reunion^ neurons are specifically modulated by social isolation and reunion, rather than general stress, salient stimuli or other physiological needs and their functions are conserved across different mouse strains. MPN^Reunion^ neurons are activated by gentle touch and send direct inhibitory input to MPN^Isolation^ neurons, indicating a circuit mechanism in which loss of social contact during isolation activates MPN^Isolation^ neurons to signal an aversive state and enhance social motivation.

Notably, the opposite functions and mutual interactions between MPN^Reunion^ and MPN^Isolation^ neurons share a striking similarity with the organization of hypothalamic circuits underlying physiological homeostasis, for example Arc^*AgRP*^ and Arc^*POMC*^ neurons controlling appetite^12,13^, and distinct neuronal populations regulating thirst^14–16^ and sleep^17^. This circuit architecture may therefore represent a common neural strategy to encode evolutionarily conserved behavioural drives in the hypothalamus, further highlighting the significance of social need for survival.

Physiological needs are monitored using peripheral signals, such as metabolite concentration for appetite^12,13^ and osmolality for thirst^14–16^. Our data indicate that touch is essential for perceiving social environment, further supporting the role of mechanosensation in mediating social motivation in rodents^43–45^ and conspecific detection in zebrafish^46^. Social isolation effectively promotes touch seeking in FVB mice, whereas isolated C57 mice lack touch preference, which may underlie their lower social rebound or, instead, reflect a limited contribution of touch to social need in that strain.

Social touch is known to trigger dopamine release in the NAc^40^, contributing to social reward^18,19^. Similarly, we observed significant dopamine release in NAc on social reunion and identified extensive projections from MPN^Reunion^ neurons to VTA, which presumably contribute to this effect. NAc neurons are activated during social reunion and project to both MPN^Isolation^ and MPN^Reunion^ neurons, indicating an MPN–VTA–NAc circuit loop that provides real-time social motivation supporting the execution of social rebound. By contrast, social isolation activates brain areas and cell types signalling negative valence, thus generating an aversive emotional state. Dopamine neurons within the dorsal raphe nucleus (DRN) have been proposed to encode the aversive state associated with social isolation^20^ and arousal to salient stimuli^47^. Our data uncover projections from MPN^Reunion^ neurons to DRN (Extended Data Fig. 9h,i), indicating possible modulation of DRN by MPN^Reunion^ neurons during social reunion.

How does the brain track the duration of social isolation and trigger a scalable social rebound after prolonged isolation? Our results indicate that MPN^Isolation^ neurons may play a key role in this process. MPN^Isolation^ neurons showed rapid activation at the onset of isolation and continuous activity over hours of isolation. Over days, both the number of MPN^isolation^ neurons and their activity strength increase as isolation progresses. Across mouse strains, more robust activation of MPN^Isolation^ neurons is associated with more pronounced social rebound. Moreover, optogenetic activation of MPN^isolation^ neurons leads to a sustained dose-dependent effect in social rebound. Our study also showed that the activity of MPN^Isolation^ neurons is further integrated in downstream neurons releasing neuropeptides or other signalling molecules, such as PVN^*Oxt*^ neurons^48^. In addition, the activity of MPN^Isolation^ neurons may facilitate synaptic plasticity of local or global neural circuits to magnify social stimuli and trigger social rebound during reunion.

Altogether, we have uncovered brain-wide circuits centred around two hypothalamic cell populations, MPN^Isolation^ and MPN^Reunion^ neurons, that monitor touch signals to provide animals with a dynamic neural representation of social environment and control the behavioural and emotional displays of social homeostasis. Insights into the nature and identity of circuits controlling social need, and the marked circuit similarity with physiological homeostatic controls, open new avenues for understanding and treating mental and physical disorders induced or exacerbated by social isolation.

## Methods

### Animals

Mice were maintained on a 12 h:12 h dark-light cycle with access to food and water ad libitum. The temperature was maintained at 22 °C and the humidity between 30% and 70%. All experiments were performed in accordance with National Institutes of Health guidelines and approved by the Harvard University Institutional Animal Care and Use Committee. Experiments were performed in adult female mice to avoid interfering behaviours such as aggression or mating seen in male mice after social isolation. The following mouse lines (strain no.) were obtained from the Jackson Laboratory: BALB/cJ (000651), DBA/2J (000671), C57BL/6J (000664), C3H/HeJ (000659), SWR/J (000689), FVB/NJ (001800), sighted FVB (FVB-Pde6b^+^, 004828), TRAP2 (also called Fos^2A-iCreERT2^, 030323), Ai9(RCL-tdT) (007909), Mc4r-2a-Cre (030759), Vglut2-IRES2-FlpO-D (030212), VGAT-2A-FlpO-D (029591), ROSA-DTA (009669) and Nav1.8-Cre (036564). We obtained *Pde6b*^*rd1*^ mutant mice in C57BL/6J background from C. Cepko (Harvard Medical School) and Mrgprb4-Cre mice from I. Abdus-Saboor (Columbia University). Trpc2 knockout mice were generated previously in our lab^36^. The following mouse lines were backcrossed to FVB/NJ mice for (*N*) generations before experiments: TRAP2/Ai9 (*N* > 9, activity labelling experiments), Vglut2-Flp and VGAT-Flp (*N* ≥ 5, neural tracing and optogenetics), Mc4r-Cre (*N* = 3, calcium imaging and optogenetics), Mrgprb4/DTA, Nav1.8/DTA and Trpc2 (*N* = 1, behavioural assay). The sample sizes for experiments were chosen on the basis of common practices in animal behaviour experiments.

### Behavioural assays

All behavioural experiments were performed during the dark cycle of the animals in a room illuminated by infrared or red light. Mice were habituated in the room for 10–20 min before experiments. For anxiety and stress tests, mice were acclimatized to the testing environment for 1 h before testing to reduce basal stress level.

#### Social isolation/reunion assay

Female sibling mice were group housed (at least three mice) after weaning for at least 1 week (4-week-old mice) before social isolation experiments. Before isolation, two group-housed cagemates were put together in a new cage with fresh bedding for 10 min to measure the baseline social interaction (isolation day 0). One mouse was then isolated in their home cage or in a new cage for 5 days while the other was kept in group. On the first, third and fifth day from the onset of isolation, the isolated mice were reunited transiently with the same group-housed cagemate in a new cage for 10 min. In each reunion session, the isolated mice were first put in the cage, and then its group-housed cagemate was introduced. All behaviours occurring during the reunion period were recorded using a multi-camera surveillance system (GeoVision GV-VMS software and GV-BX4700-3V cameras). Behaviour videos were scored manually using the Observer XT 11 software (Noldus Information Technology) to identify typical social behavioural modules, including approaching (one mouse moves towards another), sniffing (the nose of one mouse comes close to or makes contact with another mouse’s body, usually the anogenital region), crawling under (one mouse crouches down, crawls underneath another mouse’s body and sometimes passes through), head-to-head contact (two mice approach each other and contact each other’s noses) and allogrooming (one mouse grooms another mouse, usually on the head, neck and back regions). Every single event of these behavioural modules was considered as a social interaction bout, and the intervals between social interaction bouts were measured as behavioural latency. The transition probability of one behavioural module occurring after a different module was calculated across all reunion sessions to quantify the unique motor sequences of social interaction during reunion (Extended Data Fig. 1f). The total durations of all the modules within one reunion session were summed up to calculate total social interaction, and the social interactions initiated by the isolated mice were used to indicate social rebounds unless otherwise noted. To demonstrate the satiation process during social rebound, we calculated social interaction in discrete time bins (2 min per bin) during reunion. Social interactions were also measured after 1, 3, 6 and 12 h of isolation or 7 and 10 days of isolation to show the emergence of social rebound or its plateau phase. Custom MATLAB codes and DeepLabCut software package were used to track frame-by-frame positions of two mice during social reunion, and the social distances between two mice were calculated and averaged across frames during one reunion session (Fig. 1e). Because 1 and 3 days of social isolation are sufficient to trigger significant social rebound in FVB/NJ mice, we used these two isolation schedules in our functional manipulation experiments.

#### USV detection

USVs during social reunion were recorded in a sound isolation box using an ultrasonic microphone (Ultrasound Gate CM16/CMPA; Avisoft Bioacoustics) positioned 30 cm above the floor of the cage and converted into a digital format by an analogue-to-digital converter (Ultrasound Gate 116, Avisoft Bioacoustics) sampling at 500 kHz. The Avisoft Recorder software was used to control the recording and audio files were saved as 16 bit WAV format and later analyzed with DeepSqueak^50^—a deep learning-based system for detection and analysis of USVs. The built-in mouse call detecting neural network was used to identify USV syllables, and the detected syllables were reviewed manually to correct false labelling. The corrected detection files were then exported as .mat files. Custom MATLAB code was used to extract the timing and duration of each USV. The number of USVs was plotted as a function of different timecourses to show the dynamic changes within and across reunions. As only one microphone was used for recording, we were not able to determine the source of USVs; however, as group-housed female mice emit few USVs, and we detected USVs from isolated mice before cagemates were introduced, we assumed that most of the recorded USVs during reunion were probably generated by the isolated mice. Correlation between USVs and social interaction initiated by the isolated mice were assessed over time.

#### Non-social object interaction test

To test whether social isolation increases the motivation for general investigation behaviours, we tested behaviour towards a non-social object in group-housed or isolated mice. Before isolation, group-housed mice were presented with a 15 ml centrifuge tube or a rubber toy mouse in a new cage for 10 min to measure baseline behaviour. Mice were then isolated in their home cage or in a new cage for 3 days before a second test with the object. Behaviour during tests was recorded and analyzed. Any contact or climbing behaviours with the object were scored and added up as the time spent interacting with the object.

#### Social interaction in different phases of oestrous cycle

To test whether social rebound in female mice after social isolation is influenced by the oestrous stage of the animal, we performed reunion assays after 3 days of social isolation and identified the oestrous phase of each tested mouse. Time spent in social interaction was compared between mice in either oestrus or dioestrus phases. Vaginal smears were examined under the microscope, and specific oestrous stages were characterized on the basis of the morphology of vaginal epithelial cells as described previously^51^. In brief, we collected vaginal cells from female mice with 10 µl of PBS and observed these samples under a light microscope with a ×40 objective to characterize the morphology of cells. During the oestrus phase, vaginal epithelial cells are cornified and appear large and flat, while during the dioestrus phase, these cells are smaller with round shapes^51^.

#### Anxiety and stress assays

Mice’s anxiety levels were measured after different durations of social isolation using elevated plus maze and open field tests. The elevated plus maze was on a pedestal 1 m above the ground and consisted of two closed arms (30 × 5 cm^2^ with 15-cm-high wall) and two open arms (30 × 5 cm^2^) arranged 90° from each other with a central platform (5 × 5 cm^2^), all made of black acrylic board. Mice were placed on the central platform and allowed to freely explore the maze for 5 min while the behaviour was recorded. All behaviour videos were scored manually with Noldus Observer software to measure the total time spent in the open and closed arms. In the open field test, individual mice were placed in a 42 × 42 × 42 cm^3^ arena composed of black acrylic board and allowed free exploration for 10 min. The behaviour was recorded and analyzed to measure the total time spent in centre (24 × 24 cm^2^) and peripheral zones. To induce physical restraint stress, individual mice were placed into 50 ml conical tubes with ventilation holes for 1 h and then reunited with one of their cagemates in a new cage to monitor the behaviours with the same settings as described for social reunion assays.

#### Social preference tests

To examine the preference of the tested mice for a group versus a single mouse, we built a new arena with three cubic chambers (25 × 25 × 25 cm^3^) joined by a triangular central zone to allow for unbiased entry into any chamber (Fig. 1p). Each chamber contained an inverted wire cup. The cup was empty in the C0 chamber, contained one cagemate in the C1 chamber and contained three cagemates in C3 chamber. The locations of the three types of chamber were assigned randomly across animals. Group-housed or isolated mice were first introduced into the central zone at the beginning of the test and allowed to explore the arena freely for 10 min. Behaviours were recorded and time spent in each chamber was scored manually. In the preference test between unfamiliar mice versus cagemates (Extended Data Fig. 1m), a standard three-chamber task was used in which the arena consisted of two choice chambers on two sides and a central zone that allowed for unbiased entry into any chamber. Each of the two choice chambers contained an inverted wire cup with either a familiar mouse (cagemate) or a stranger from the same strain. The tested mice (group-housed or isolated) were first introduced into the central zone at the beginning of the test and allowed to explore the arena freely for 10 min. Behaviour was recorded and time spent in each chamber was scored. The same arena and experimental procedure were used in the strain preference test, in which the two choice chambers contained mice from either the same or different strains.

#### Sensory modality screening

To investigate the contribution of different sensory modality to the emergence of social rebound after isolation, we designed a home-cage divider experiment in which a plastic divider with laser-cut thin slots/openings was placed in the diagonal of home cage to subdivide a group of three mice, such that one mouse was placed into one side and the other two together on the other side. Food, water and nesting materials were provided equally on both sides. The divider prevented the separated mice from physically interacting with each other but allowed exchange of auditory and olfactory information. After 3 days of separation, the singly divided mouse was reunited with one of the mice from the other side in a new cage for 10 min. The occurring behaviours were recorded and scored manually. The time spent in social interaction was compared with the social rebound after 3 days of isolation in the singly housed condition. To examine the potential contribution of pheromone sensing to social rebound, we assessed the behaviour of *Trpc2*^−/−^ mice, which are impaired in vomeronasal pheromone sensing. *Trpc2*^−/−^ mice were first crossed to FVB/NJ strain for one generation, and the resulting *Trpc2*^+/−^ mice were used to cross to each other to generate wild-type (*Trpc2*^+/+^) and mutant (*Trpc2*^−/−^) mice for the experiments. We measured the rebound social interaction after 3 days of isolation and analyzed the satiation process in wild-type and mutant mice.

#### Gentle touch preference test

To test the preference of gentle touch before and after social isolation, we designed a free choice task for mice to interact with either a naked plastic tunnel (10-cm long, provided by Harvard Biological Research Infrastructure) or a tunnel lined inside with a layer of soft plush towel (bought from Amazon), referred to as cloth tunnel. All materials were autoclaved before use and replaced between animals to avoid odour contamination. During the test, group-housed or 1-day isolated mice were placed in a new cage with one naked tunnel and one cloth tunnel. Mice were allowed to explore freely and go through either tunnel for 15 min. Behaviours were recorded, and the crossing events were scored. The touch preference indexes were measured in terms of the number or the duration of crossings through the cloth tunnel divided by all crossings through both tunnels.

#### Touch manipulation assays

To acutely reduce tactile sensitivity during social reunion, isolated mice received an intraperitoneal injection of isoguvacine^37^ (20 mg kg^−1^)—a peripherally restricted GABA_A_R agonist—60 min before social reunion. PBS was injected in a different batch of isolated mice, which served as a control group. To examine the contribution of tactile sensation to the emergence of social need, we genetically ablated somatosensory neurons marked by Mrgprb4 or Nav1.8, which are thought to mediate social touch in mice^39–41^. We first separately backcrossed Mrgprb4-Cre (B4-Cre), Nav1.8-Cre and Cre-dependent DTA mouse lines (all in C57BL/6J background) to FVB/NJ strain for one generation and then crossed the resulting F1s (F1(B4-Cre/FVB), F1(Nav1.8-Cre/FVB) and F1(DTA/FVB)) to ablate Mrgprb4-lineage neurons (B4/DTA) or Nav1.8^+^ neurons (Nav1.8/DTA). We measured social interaction in B4/DTA and Nav1.8/DTA mice after 0 or 3 days of isolation and compared these results with the social rebound measured in wild-type mice from FVB/NJ × C57BL/6J cross. In the acute touch rescue experiments (Fig. 5h), faux-fur-lined tunnels were used to provide comfort-touch stimulation as an enhanced version of cloth tunnel described in ‘Gentle touch preference test’. Mice were habituated to the faux-fur-lined tunnels by continuously going through two of these tunnels that were alternatingly connected by experimenter before social isolation. Mice were then isolated for 24 h and reunited with one cagemate both before and after gentle touch stimulation. Specifically, a 5-min reunion assay was first performed to measure the baseline social interaction, and then the mice went across faux-fur-lined tunnels 30 times the same way as in habituation. The 30 crossings typically took around 10 min for both cloth tunnel and naked control tunnel. A second reunion assay was then conducted to measure the change of social motivation compared with the first reunion. Naked plastic tunnels were used in another batch of animals as a negative control. In the chronic touch rescue experiment (Extended Data Fig. 10e), isolated mice were co-housed with either a cloth tunnel or a naked tunnel described in ‘Gentle touch preference test’ for 24 h. A social reunion assay was then carried out to measure and compare social interaction after different co-housing conditions.

### Microendoscopy calcium imaging

To examine real-time neuronal activity with single-cell resolution in the MPN, we performed microendoscopy calcium imaging in FVB/NJ (*n* = 6), C57BL/6J (*n* = 6) and Mc4r-Cre/FVB mice (*n* = 3). The Mc4r-Cre mouse line, used for labelling MPN^Isolation^ neurons, was backcrossed for at least three generations to FVB/NJ strain before experiments. All imaging experiments were performed during the dark cycle of the animals in a chamber illuminated by infrared light.

#### Virus injection and GRIN lens implantation

For pan-neuronal activity imaging, 400 nl of AAV1-Syn-GCaMP6s (Addgene, catalogue no. 100843-AAV1) was injected unilaterally into the MPN of FVB/NJ or C57BL/6J mice, at anterior-posterior 0, medial-lateral 0.3 and dorsal-ventral −4.8 (Paxinos and Franklin atlas). To image MPN^*Mc4r*+^ neurons, 400 nl of AAV1-Syn-Flex-GCaMP6s (Addgene, catalogue no. 100845-AAV1) was injected unilaterally into the MPN of Mc4r-Cre/FVB mice using the above coordinates. As the brain anatomy of FVB/NJ strain differs slightly from the Paxinos and Franklin brain atlas, we adjusted the anterior-posterior coordinate 0.4–0.5 mm towards the rostral side to target the MPN in FVB/NJ mice. At 30 min after viral injection, a 25-gauge blunt needle (SAI infusion technologies, VWR, catalogue no. 89134-146) was slowly inserted (1 mm per 5 min) into the brain targeting anterior-posterior 0, medial-lateral 0.3 and dorsal-ventral −4.7 (Paxinos and Franklin atlas) to create a tract. The needle then was withdrawn slowly, and a GRIN lens (Inscopix, 0.6 × 7.3 mm) was inserted slowly (1 mm per 10 min) into the tract formed by the needle and targeted at 100 µm below the end of the needle tract. The lens assembly with the baseplate was secured on the skull with dental cement (Parkell), and a titanium headplate was attached at the base of the lens assembly with dental cement to restrict the animal’s head for attaching to the microendoscope. The positions of all implanted GRIN lenses were assessed using post hoc histology and only the imaging data from correctly targeted lens were used for further analysis. Mice were housed individually after surgery for 1 week and then co-housed with one or two cagemate(s) for another 3–4 weeks to allow for the expression of GCaMP and clearing of the imaging window.

#### Calcium imaging and behavioural assays

Optimal imaging settings (focal and excitation parameters) were determined on the first day of the imaging experiments. The implanted mouse was restrained transiently and the microendoscope (nVista3, Inscopix) was attached onto the lens assembly on the head of the mouse. The focusing plane of the microendoscope was adjusted carefully over the entire working distance to choose an imaging plane with most neurons and sharp image. The field of view was cropped to the region encompassing the entire lens field of view. The illumination power (roughly 10% of the maximum power) and the sensor gain (roughly 10–20% of the maximum gain) were chosen to have a strong relative increase in fluorescence signal, but not saturated. These imaging settings were saved for each animal and used subsequently for the same animal across sessions. We used inbuilt acquisition software from Inscopix to acquire images at 10 Hz. Before formal data acquisition, mice were habituated to the recording setup and environment two to three times. An Arduino microcontroller is programmed to send transistor–transistor logic (TTL) signals to synchronize the data acquisitions of calcium signals (DAQ, Inscopix), animal behaviour recoding (BFS-U3-31S4M-C, FLIR Blackfly S camera) and USV recoding (Avisoft Bioacoustics). In the social reunion assays, implanted mice were isolated for 0, 1, 3 or 5 days before imaging. During the imaging sessions, there was an initial 5- or 10-min baseline period during which the mouse was kept alone. Following this, a cagemate was introduced into the cage, and social interaction between the mice was allowed for 5 or 10 min. Subsequently, the cagemate was removed, and the mouse was kept alone again for another 5 or 10 min. To monitor the neuronal activity during the initial 6 h of social isolation but avoid GCaMP signal photobleaching, we performed 15 min of imaging every hour for 6 h with the microendoscope connected during the whole 6-h procedure. This allowed us to track the same neurons across 6 h of isolation. We provided food and hydrogel in the cage to ensure that the mice did not experience hunger or thirst. At the end of the 6-h imaging, we performed a 5-min reunion assay to identify neurons with significantly modulated activity. To examine the activity specificity, we performed a series of control experiments in which a 3-day isolated mouse encountered (for 10 min) one control stimulus, including food pellets, a C57 stranger female, a pup and a castrated male. Then we removed the control stimulus and left the mouse alone for a 15-min interval, followed by a 5-min reunion assay to identify the significantly modulated neurons. During the whole procedure, the microendoscope was connected to the animal to track the same neurons across different events. Distinct stimulation was presented in separated imaging sessions with a random order to avoid the influence across stimulations. We used a castrated male to provide social companion and avoid mating or aggressive behaviours. Before imaging with food pellets, the mouse was food restricted overnight. Tail suspension was performed by suspending the mouse manually by the tail for up to 2 min and usually after reunion to avoid its stressful influence on social behaviour. In the tunnel crossing experiment, the implanted mouse was isolated for 3 days before imaging. During the imaging session, there was a 5-min baseline period followed by 15 min of free tunnel crossing during which two faux-fur-lined tunnels (10 cm long, 7.5 cm in diameter, the fur around 1.5 cm long) were introduced into the cage to maximize crossing behaviours. The top of the tunnel was removed creating a slot (4 cm) to allow the implanted mice to run through with the attached wire. The tunnels were subsequently removed from the cage and the mouse was kept alone for 30 min. Finally, we performed a 10-min imaging with 5 min of baseline and 5 min of reunion to identify MPN^Isolation^ and MPN^Reunion^ neurons.

#### Image processing and calcium signal extraction

The images acquired were processed in two steps. In the first step, we processed the raw image data using the Inscopix data processing software (IDPS v.1.8.0.3519). The images were imported in the proprietary Inscopix format to IDPS. The images were spatially downsampled by a factor of four to reduce the file sizes for subsequent steps without losing quality, followed by a spatial bandpass filter in the frequency band of 0.005 to 0.5 per pixel. The images were next subjected to motion correction using IDPS and the resulting images were saved as a tiff image stack. The second step used standard MATLAB scripts from the CNMFe^52^ database (https://github.com/zhoupc/CNMF_E). Calcium traces were extracted and deconvolved using CNMFe pipeline with the following parameters: patch_par = [2,2], gSig = 3, gSiz = 13, ring_radius = 9, min_corr = 0.8, min_pnr = 8, deconvolution: foopsi with the ar1 model^52^. The spatial and temporal components of every extracted unit were inspected carefully manually (SNR, PNR, size, motion artifacts, decay kinetics, and so on) and outliers (obvious deviations from the normal distribution) were discarded. Cells with elongated and thin shapes were removed using custom MATLAB codes.

#### Single-neuron and population activity analysis

All analysis based on extracted calcium traces were performed using custom MATLAB scripts. To categorize the pan-neuronal calcium activity patterns at single-neuron level during social reunion, reunion induced responses were calibrated for each neuron using ROC analysis as described previously^28,53^. ROC curves were calculated by comparing the distribution of raw calcium responses during baseline (isolation, 300 s) and during reunion (300 s) (Extended Data Fig. 2a,b). A series of thresholds moving from the minimum calcium response to the maximum were set to evaluate the binary separation of the calcium activity from baseline versus reunion. For each threshold, we calculated the probability that the baseline activity was greater than the threshold (falsely categorized as reunion activity, that is, false positive rate) and the probability that reunion activity was greater than the threshold (correctly categorized as reunion activity, that is, true positive rate). An ROC curve was then plotted by graphing the true positive rate against the false positive rate for all thresholds. The area under the ROC curve (AUC) was used to quantify the response strength of each neuron. AUC values more than 0.5 indicate increased response compared with baseline, whereas AUC values less than 0.5 indicate decreased response. The significance of the responses was determined by comparing AUC from real data and randomly shuffled data. Specifically, we randomly shuffled the calcium activity in each time bin (1 s) between baseline and reunion period 1,000 times and calculated the AUC values in each shuffle. Neurons with the AUC value >0.7 or <0.3 and exceeded the 95th percentile of the AUC distribution from shuffled data, were considered significantly tuned by reunion, among which the inhibited neurons are referred to as MPN^Isolation^ neurons and the activated neurons are referred to as MPN^Reunion^ neurons. We used the AUC values to infer the activation strength of MPN^Reunion^ neurons during reunion and (1 − AUC) values to infer the (relative) activation strength of MPN^Isolation^ neurons during isolation (baseline) period. In the imaging experiments during the initial 6 h of isolation, neurons with the activity that met the above standard for at least 1 h were identified as MPN^Isolation^ neurons. Similar ROC analyses were applied to the neuronal responses during other imaging experiments. The total duration of activation or inhibition for each neuron during reunion was calculated by counting the time bins where calcium activity was modulated significantly, either above 2  s.d. from the baseline mean or below 30% of the baseline mean. The duration values from each neuron were averaged to obtain the mean for the animal (Extended Data Fig. 2h). The persistent activation or inhibition for each neuron was quantified as the longest duration where the calcium activity in all time bins was modulated significantly, and the duration values from each neuron were averaged to obtain the mean for the animal (Extended Data Fig. 2i). To visualize the population activity during social reunion assay, we performed PCA with significantly tuned neurons from all imaged mice with the same isolation schedule and projected their activity (*z* scores) onto the first three principal components to visualize the neural trajectory across time (bin size, 5 s; Fig. 2g). To quantify state changes, the Euclidean distances between each point in the trajectory (time bin, 1 s) and the mean of the baseline were calculated using the minimum number of principal components that explain 95% of the variance. This same procedure was repeated 100 times by selecting 50% of the neurons randomly from the imaging sessions to calculate the mean and s.d. (Fig. 2h). To describe how the isolation state is re-established during the re-isolation period, when the partner mouse was removed after a brief reunion, the latency for the PCA distances to return to the initial isolation (baseline) state and the durations after return were calculated using the upper bound of the 95% confidence interval of the baseline as a threshold (Extended Data Fig. 3a). In the population activity decoding analysis of isolation versus reunion states, we trained support vector classifiers using 80% of the data that were selected randomly from each state and tested the accuracy of the classifiers with the remaining 20% data (Extended Data Fig. 3b). To identify the neurons with significant ramp-up or ramp-down activity during the initial 6 h of isolation, calcium activity averaged in 5-min bins was used to run a linear correlation test against isolation durations using the ‘corrcoef’ function in MATLAB, and the neurons with *P* values < 0.05 were considered to have significant ramp-up or ramp-down activity (Extended Data Fig. 2j).

In cell-type targeted imaging experiments, we monitored the calcium activity of MPN *Mc4r*^+^ neurons from three mice in FVB background, identified significantly inhibited neurons during reunion and examined their activity during other behavioural paradigms, including eating and tail suspension. To identify the same neurons across imaging sessions, we aligned the fields of view from different imaging experiments. If the overlaps between neuron pairs were higher than 70%, these neurons were considered the same. Alternatively, for the experiments performed on the same day (Fig. 5i and Extended Data Fig. 3c–h), the raw imaging data from two sessions in the same field of view were concatenated together to extract calcium traces.

### Cell-type identification

#### TRAP induction and activity labelling

To specifically label neurons activated during social isolation, we took the advantage of the TRAP2/Ai9 mouse line^16^ that enables the labelling of activated neurons over a 3–6 h time window, which better integrates neuronal activity representing a persistent state compared with *Fos* in situ hybridization. The TRAP2/Ai9 line was backcrossed to FVB/NJ strain for more than nine generations before use. The isolation-activated neurons were ‘TRAPed’ in the following steps: 10 mg of 4-OHT (Sigma Aldrich, catalogue no. H6278-10mg) was added with 500 μl ethanol and shaken at room temperature until the powder was completely dissolved (20 mg ml^−1^). The resolved 4-OHT solution was then mixed with corn oil in 1:2 volume ratio and vortexed to fully mixed to extract the 4-OHT in the oil. The resulting solution was vacuum centrifuged for 1–1.5 h until the upper layer of ethanol was evaporated. The final 10 mg ml^−1^ 4-OHT solution was injected intraperitoneally into mice immediately after preparation at a dose of 50 mg kg^−1^. To label (‘TRAP’) the cells activated during social isolation, mice were isolated for 3 days and injected with the 4-OHT solution in the dark phase of the third isolation day. After at least 7 days, the mice were euthanized for in situ hybridization experiments. To assess the activity of PBN during isolation, we performed TRAP inductions after 1 or 3 days of isolation in different cohorts of animals. To label the neurons that are activated during social reunion, FVB/NJ mice were isolated for 3 days and reunited with one of its cagemate. At 30–40 min after the onset of the reunion, mice were euthanized for in situ hybridization experiments. The reunited mice were kept with cagemate from reunion assay until euthanasia to prevent the activation of isolation-related neurons. To assess the activity of PBN during reunion, 4-OHT was injected 1 h after reunion and the reunited mice were kept in group for at least 24 h to allow tamoxifen to be fully metabolized.

#### In situ hybridization

RNAscope v.2 kit (Advanced Cell Diagnostics (ACD)) was used to perform double-label and triple-label fluorescence in situ hybridization according to the manufacturer’s instructions. Probes for *tdTomato* and *Fos* were used to visualize activated neurons labelled with TRAP method or acute behavioural assays, respectively. Probes for marker genes of specific cell types were selected from previous single-cell RNA sequencing and functional studies. All probes were made by ACD. Animals were euthanized after specific behavioural assays and the brains were dissected. Freshly frozen brains were sectioned using a cryostat at 16 μm and stored at −80 °C. On the day of the fluorescence in situ hybridization experiment, slides were thawed and fixed in 4% paraformaldehyde (PFA) for 15 min followed by dehydration in 50%, 75% and 100% ethanol at room temperature. Tissue samples were processed using 3% hydrogen peroxide (VWR) for 10 min and permeabilized for 25 min using Protease IV (ACD). For each RNAscope experiment, C2 and C3 probes were diluted in C1 probe solution (1:50), heated to 40 °C for 10 min and applied to slides that were placed in ACD HybEZ II oven at 40 °C for 2 h. Tissue samples were then processed as suggested by the RNAscope v.2 protocol (ACD). Slides were imaged at ×10 on an Axioscan 7 using Zen Blue v.3.5 software (Zeiss). The number of cells marked by specific and overlapping genes was measured using QuPath v.0.3.2.

### Neural circuit tracing

#### Anterograde tracing

Anterograde tracing experiments were performed in TRAP2/Vglut2-Flp mice and TRAP2/Vgat-Flp mice both in FVB/NJ background. TRAP2, Vglut2-Flp and Vgat-Flp lines were backcrossed separately to FVB/NJ strain for more than five generations and the offspring from these lines were crossed to generate TRAP2/Vglut2-Flp/FVB mice for tracing experiments of MPN^Isolation^ neurons and TRAP2/Vgat-Flp/FVB mice for tracing experiments of MPN^Reunion^ neurons. All surgeries were performed under aseptic conditions in animals anaesthetized with 100 mg kg^−1^ ketamine and 10 mg kg^−1^ xylazine by intraperitoneal injection. Using a programmable nano-injector (Nanoject III, Drummond), 150–200 nl of Cre- and Flp-dependent virus AAV8-hSyn-Con/Fon-EYFP (Addgene, catalogue no. 55650-AAV8) was injected unilaterally into the MPN (anterior-posterior 0, medial-lateral 0.3 and dorsal-ventral −5, Paxinos and Franklin atlas). We adjusted the anterior-posterior coordinate 0.4–0.5 mm towards the rostral side to match the anatomy of the FVB/NJ brain. After surgery, injected mice were housed singly to recover for 1 week and then put together with former cagemates for another week before TRAP induction (see details in ‘TRAP induction and activity labelling’). To visualize the projections of MPN^Isolation^ neurons, TRAP2/Vglut2-Flp/FVB mice were isolated for 3 days and injected with 4-OHT. To visualize the projections of MPN^Reunion^ neurons, TRAP2/Vglut2-Flp/FVB mice were isolated for 3 days and injected with 4-OHT 1 h after reunion. After 2 weeks of viral fluorophore expression, animals were perfused transcardially with PBS followed by 4% PFA in PBS. Brains were dissected and post-fixed in 4% PFA overnight. After embedding in 4% low-melting point agarose (Promega, catalogue no. V2111) in PBS, 50-μm coronal sections were cut through the whole brain on a vibratome (Leica) and mounted on slides (VWR, catalogue no. 48311-703) with DAPI-containing mounting medium (Vector Laboratories, catalogue no. H-1200). The brain sections were imaged at ×10 magnification using AxioScan 7 and Zen Blue v.3.5 software (Zeiss). For quantification of projection density, the average pixel intensity in a target region containing EYFP signals was calculated, and the background was subtracted (Zen Blue software, Zeiss). Because injections were unilateral and no labelling was observed in most cases contralaterally, the equivalent region on the contralateral hemisphere was chosen for background subtraction; in cases where contralateral EYFP were present, an adjacent unlabelled region was chosen. The relative density value for each projection region was calculated as the ratio between background-corrected intensities in each region divided by the sum across all the target regions.

#### Monosynaptic retrograde tracing

Monosynaptic retrograde tracing experiments were performed with an intersectional strategy in TRAP2/Vglut2-Flp/FVB mice and TRAP2/Vgat-Flp/FVB mice (see details in ‘Anterograde tracing’). We first injected 150–200 nl of a 1:1 mixture of two Cre- and Flp-dependent viruses, AAV8-nEF-Con/Fon-TVA-mCherry (Stanford GVVC-AAV-197) and AAV8‐Ef1a‐Con/Fon-oG (Stanford GVVC-AAV-198) unilaterally into the MPN with the same coordinates as in ‘Anterograde tracing’. The injected mice were housed singly for 1 week and reunited with former cagemates for another week before TRAP induction (see details in ‘TRAP induction and activity labelling’). TRAP2/Vglut2-Flp/FVB mice were isolated for 3 days and injected with 4-OHT to allow viral expression in MPN^Isolation^ neurons; TRAP2/Vgat-Flp/FVB mice were isolated for 3 days and injected with 4-OHT 1  h after reunion to enable viral expression in MPN^Reunion^ neurons. Two weeks later, 200 nl of G-deleted rabies virus (EnvA-ΔG-rabies-eGFP, Janelia Viral Tools Facility) was injected into the MPN. Seven days later, mice were euthanized, and the brains were dissected, sectioned and imaged with the same procedure as in ‘Anterograde tracing’. Relative input strength was quantified as follows. First the representative sections of input regions were selected and GFP^+^ cells (presynaptic cells) were counted. The local presynaptic cells in the MPN were estimated by counting GFP^+^ and mCherry^−^ neurons. The relative input density was calculated as the ratio between number of presynaptic cells in each input region divided by the sum across all calculated regions in each brain. To identify the input cell types of MPN^Isolation^ neurons, a different cohort of mice were processed for in situ hybridization (see details in ‘In situ hybridization’). Probes for the *GFP* gene, marker genes or immediate early genes were used to examine the presynaptic cell types.

### Optogenetics

#### Virus injection and fibre implantation

TRAP2/Vglut2-Flp mice and TRAP2/Vgat-Flp mice were injected bilaterally with 200 nl of AAV8-hSyn Con/Fon-hChR2(H134R)-EYFP for optogenetic activation (Addgene catalogue no. 55645) or AAV8-nEF-Con/Fon-iC++-EYFP for optogenetic inhibition (Addgene, catalogue no. 137155) into the MPN (anterior-posterior 0, medial-lateral ±0.3 and dorsal-ventral −5, Paxinos and Franklin atlas) and in the same surgery a dual fibre-optic cannula (200/250-0.66_GS0.6/0.8_FLT, Doric Lenses) was implanted 200 µm above the injection site for MPN cell body manipulation or above the Arc (anterior-posterior −1.6, medial-lateral ±0.3 and dorsal-ventral −5.3) or the LHb (anterior-posterior −1.6, medial-lateral ±0.3 and dorsal-ventral −2.2) for projecting axon terminal manipulation. To manipulate MPN *Mc4r*^+^ neurons, 200 nl of AAV-EF1a-DIO-hChR2(H134R)-EYFP (UNC Vector Core) or AAV-EF1a-DIO-iC++-EYFP (UNC Vector Core) was bilaterally injected into the MPN, and the dual fibre-optic cannula was implanted. Mice were recovered for 2 weeks before Isolation-TRAP and Reunion-TRAP induction (see details in ‘TRAP induction and activity labelling’). Mice were tested 3–5 weeks after TRAP induction to allow for efficient expression of ChR2 or iC++.

#### Optogenetic manipulations

On testing days, the implanted optic fibres were attached through a patch cord (SBP(2)_200/230/900-0.57_FCM-GS0.6/0.8, Doric Lenses) and a rotary joint (FRJ_1 × 1_FC-FC, Doric Lenses) to a 460-nm blue LED module (Prizmatix) for optogenetic activation or inhibition. An Arduino microcontroller is programmed to send TTL signals to a light-emitting diode (LED) module to control the stimulation patterns. Pilot experiments were conducted to test and determine the proper ranges of LED power in different manipulation experiments. After connected to the patch cord, mice were transferred to a new cage with the same setup in social reunion assay and allowed to habituate for 5–10 min. To activate Vglut2^+^/Isolation-TRAPed neurons or Mc4r^+^ neurons, the LED was on for 1 s (20 Hz, 10 ms pulses, 6–8 mW at patch cord tip) and off for 3 s, repeatedly. To activate Vgat^+^/Reunion-TRAPed neurons, the LED was on for 40 s (20 Hz, 20 ms pulses, 6–8 mW) and off for 20 s, repeatedly. In the real-time place preference/avoidance tests, the LED was turned on (20 Hz, 6–8 mW) during the period when mice entered the LED-on chamber assigned randomly in each session. To inhibit Vglut2^+^/Isolation-TRAPed neurons or Mc4r^+^ neurons or Vgat^+^/Reunion-TRAPed neurons, the LED was on for 3 min (constant on, 3–4 mW) and off for 20 s, repeatedly. In the social interaction/reunion tests and three-chamber social preference tests, the patterned LED was applied for 10 min and the behaviours during the entire period were recorded and analyzed as the LED-on performance. In LED-off sessions, the same cohorts of animals were tested without LED stimulation. To minimize potential influence from previous experiments, we usually performed LED-off sessions before LED-on sessions with at least 3–5 days of interval between two different paradigms. Two to three batches of animals were prepared to repeat the results with a randomly assigned order of paradigms to control possible cross-paradigm influence. In the pre-reunion activation experiments, the patterned LED was applied for 5, 10 or 20 min when the animal was alone, followed by a 10-min reunion test with LED off. In the pre-reunion inhibition experiment, the LED was applied for 10 min before reunion. The real-time place preference/avoidance tests lasted for 10 min, and the patterned LED was applied when mice entered the LED-on chamber. For axon terminal manipulation experiments, we used the same protocols as in soma stimulation experiments described above. When testing the stimulation effects on food intake, mice were fasted overnight before experiments. Two food pellets were placed on the two sides of the cage and contacting one pellet triggered the LED on for 10 s whereas contacting the other pellet did not trigger stimulation treated as off controls. Weight reduction of each pellet at the end of a 10-min test was measured as the amount of food intake.

### Assessment of oxytocin system

#### Chemogenetic manipulations of oxytocin neurons

Stereotaxic surgery was used to bilaterally inject 300 nl of AAV8-hSyn-DIO-hM3D(Gq)-mCherry or AAV8-hSyn-DIO-hM4D(Gi)-mChery into the PVN of Oxt-Cre mice using the coordinates medial-lateral ±0.3, anterior-posterior −0.55, dorsal-ventral −4.9. Three weeks after viral injection, mice were used for behavioural tests. In the activation experiments, mice were isolated for 24 h and received intraperitoneal injections of CNO (0.5 mg kg^−1^) twice: once at the time of isolation and again 9 h before reunion. In the inhibition experiments, mice were isolated for 3 days and received injections of CNO four times: at the time of isolation and once each day thereafter. The final injection was administered 6–9 h before reunion to ensure that the manipulation influenced only the isolation period. Behaviours during reunion were recorded and analyzed as described above.

#### Oxytocin receptor antagonist

Mice were isolated for 24 h and received intraperitoneal injections of the oxytocin receptor antagonist (OTR-A), L-368,899 hydrochloride (5 mg kg^−1^) or saline twice as previously described^18^ first at time of isolation and again 9 h before reunion. Behaviours during the reunion period were recorded and analyzed as described above.

### Assessment of dopamine release

#### Fibre photometry

All surgeries were performed under aseptic conditions with animals anesthetized with isoflurane (1–2% at 0.5–1.0 l min^−1^). Analgesia was administered pre- (buprenorphine, 0.1 mg kg^−1^, intraperitoneal) and post-operatively (ketoprofen, 5 mg kg^−1^, intraperitoneal). We used the following coordinates to target the NAc: anterior-posterior 1.45–1.78, medial-lateral 1.0–1.4 and dorsal-ventral −3.6 to −4.1 from dura. To express dopamine sensor GRAB_DA2m_^34^, we injected 300 nl of mixed (3:1) virus solution: AAV9-Syn-GRABDA2m (Vigene Bioscience) and AAV5-CAG-tdTomato (UNC Vector Core) unilaterally into the NAc. We then implanted an optic fibre (400 µm diameter, Doric Lenses) slightly above the virus injection site. The implanted mice were housed singly for 1 week to recover and co-housed with previous cagemates for another week before photometry recording. Photometry recording was performed as previously reported^54,55^. Before recording, we connected a magnetic patch cord (400 µm diameter, numerical aperture 0.48, 3 m long, SMA-SMC, Doric Lenses) to the optical fibre implanted on the head of the animal and the animal was allowed to habituate in a new cage for 10–15 min. Once the recording started, the patch cord simultaneously delivered excitation light at different wavelength (473 nm, Laserglow Technologies; 561 nm, Opto Engine LLC) and collected fluorescence emissions from dopamine sensor and tdTomato (used for motion correction). The emitted light was then filtered using a 493/574 nm beam splitter (Semrock), followed by a 500 ± 20 nm (Chroma) and 661 ± 20 nm (Semrock) bandpass filter, and collected by a photodetector (FDS10 × 10 silicone photodiode, Thorlabs) connected to a current preamplifier (SR570, Stanford Research Systems). This preamplifier outputs a voltage signal that was collected by a data acquisition board (NIDAQ, National Instruments) and custom software written in Labview (National Instruments). Lasers were turned on at least 30 min before recording to allow them to stabilize. Before each recording session, laser power and amplifier settings were adjusted individually for each mouse. The photometry recording and behaviour video acquisition were synchronized using a common TTL input to trigger infrared light pulses (once every 10 s) that were recorded in the behaviour videos. The implanted mice were isolated for 3 days before recording. The recording session lasted for 10 min, including a 5-min baseline period where the animal was kept alone, followed by a 5-min social reunion period where a previous cagemate was introduced.

#### Fibre photometry data analysis

The tdTomato signal was subtracted from dopamine sensor signal to correct the motion artifacts. The corrected signal was then *z* scored using the mean and s.d. from a 30-s baseline period before reunion. Individual traces from different mice were aligned at the reunion time point and averaged across animals. The behaviours during recording sessions were scored manually and synchronized with photometry signals by common TTL pulses (‘Fibre photometry recording’).

#### Dopamine transporter inhibitor infusion

The infusion cannula was prepared according to a previously published protocol^56^. The cannula was implanted to the NAc bilaterally (anterior-posterior 1.45, medial-lateral 1.4 and dorsal-ventral −3.1 from dura for the guide cannula so that the plug tip/infusion needle was located at dorsal-ventral −4.1) using adhesive cement (C&B Metabond, Parkell) and a small amount of rapid-curing epoxy (Devcon, catalogue no. A00254) together with headplate. The animal was headfixed briefly to connect the infusion needle and released in the home cage. Either dopamine transporter inhibitor (5 mg ml^−1^; GBR12909, D052, Sigma Aldrich) dissolved in distilled water with 5% dimethyl sulfoxide (Thermo Fisher Scientific, catalogue no. 20688) or vehicle (distilled water with 5% dimethyl sulfoxide) was infused (300 nl per hemisphere with 200 nl min^−1^ flow rate) with a syringe pump (SP2001, World Precision Instruments) in the home cage. The mice were moved to the reunion cage 5 min after completing infusion and waited for another 5 min before reunion. Behaviours during the reunion period were recorded and analyzed as described above.

### Statistics and reproducibility

Data were processed and analyzed using MATLAB and GraphPad Prism v.9. The sample sizes for measuring social rebound in different mouse strains were determined by a MATLAB command ‘sampsizepwr’, which calculates the required cohort sizes to achieve a specified power level (0.8) necessary to confidently detect significant social rebound. In other experiments, the sample sizes were chosen on the basis of common practices in animal behaviour experiments. Individual data points were plotted wherever possible. Error bars and shaded areas in the graphs indicate the mean ± s.e.m. unless otherwise noted. All data were analyzed with two-tailed non-parametric tests unless otherwise noted. In the experiments with paired samples, we used the Wilcoxon matched-pairs signed-rank test or Friedman test. In the experiments with non-paired samples, we used the Mann–Whitney *U-*test or Kruskal–Wallis test. *P* values were corrected for multiple comparisons when necessary. Statistical significance is indicted by *P < 0.05; **P < 0.01; ***P < 0.001; NS, no statistical significance. Statistical details are given in the respective figure legends. All behavioural, imaging, in situ hybridization, optogenetics and tracing experiments were replicated in several batches of animals independently with similar results. Experiments were randomized whenever possible. Experimenters were blind to the mouse identity in Mrgprb4-lineage neuron ablation experiments.

### Reporting summary

Further information on research design is available in the Nature Portfolio Reporting Summary linked to this article.

## Online content

Any methods, additional references, Nature Portfolio reporting summaries, source data, extended data, supplementary information, acknowledgements, peer review information; details of author contributions and competing interests; and statements of data and code availability are available at 10.1038/s41586-025-08617-8.

## Supplementary information

Reporting Summary

Supplementary Video 1 Social interaction before and after social isolation, and example behavioural modules in FVB/NJ mice. Isolated mice show rebound social interaction upon reunion, while group-housed mice show sated social motivation. These video clips illustrate typical behavior modules during social reunion, that is, approaching, sniffing, crawling under, head-to-head contact and allogrooming.

Supplementary Video 2 Microendoscopy calcium imaging of MPN neurons in FVB/NJ mice during social reunion. Examples of simultaneously imaged MPN neurons (right) showing activation or inhibition upon social reunion (left).

Supplementary Video 3 Comfort-touch preference assay. Isolated but not group-housed FVB/NJ mice show increased preference to cross a cloth-lined tunnel, rather than a naked tunnel.

## Data Availability

All data that support the findings of this study are either present in figures and extended data, or available from the Dulac lab GitHub database (https://github.com/DulacLabHarvard/SocialNeed), or from the corresponding author upon request.

## Code availability

Custom codes written for behavioural and imaging data analysis are available at the Dulac Lab GitHub database (https://github.com/DulacLabHarvard/SocialNeed) or from the corresponding author upon request.
