# Supplementary material for: A hypothalamic circuit underlying the dynamic control of social homeostasis
Source: Nature. 2025 Feb 26;640(8060):1000–10. doi: 10.1038/s41586-025-08617-8 (PMC12018270; doi:10.1038/s41586-025-08617-8)
Supplement: Supplementary file 1 — Reporting Summary [file 41586_2025_8617_MOESM1_ESM.pdf]

nature portfolio

Corresponding author(s): Catherine Dulac

Last updated by author(s): Oct 24, 2024

Reporting Summary

Nature Portfolio wishes to improve the reproducibility of the work that we publish. This form provides structure for consistency and transparency in reporting. For further information on Nature Portfolio policies, see our Editorial Policies and the Editorial Policy Checklist.

Statistics

For all statistical analyses, confirm that the following items are present in the figure legend, table legend, main text, or Methods section.

n/a	Confirmed	
☐	☒	The exact sample size (*n*) for each experimental group/condition, given as a discrete number and unit of measurement
☐	☒	A statement on whether measurements were taken from distinct samples or whether the same sample was measured repeatedly
☐	☒	The statistical test(s) used AND whether they are one- or two-sided<br>*Only common tests should be described solely by name; describe more complex techniques in the Methods section.*
☐	☒	A description of all covariates tested
☐	☒	A description of any assumptions or corrections, such as tests of normality and adjustment for multiple comparisons
☐	☒	A full description of the statistical parameters including central tendency (e.g. means) or other basic estimates (e.g. regression coefficient) AND variation (e.g. standard deviation) or associated estimates of uncertainty (e.g. confidence intervals)
☐	☒	For null hypothesis testing, the test statistic (e.g. *F*, *t*, *r*) with confidence intervals, effect sizes, degrees of freedom and *P* value noted<br>*Give P values as exact values whenever suitable.*
☒	☐	For Bayesian analysis, information on the choice of priors and Markov chain Monte Carlo settings
☒	☐	For hierarchical and complex designs, identification of the appropriate level for tests and full reporting of outcomes
☒	☐	Estimates of effect sizes (e.g. Cohen's *d*, Pearson's *r*), indicating how they were calculated

Our web collection on statistics for biologists contains articles on many of the points above.

Software and code

Policy information about availability of computer code

Data collection	GeoVision GV-VMS system for behavior video acquisition,<br>Avisoft Bioacoustics for ultrasound recording,<br>Inscopix data acquisition software for microendoscope recording,<br>Zen Blue software (Zeiss) for fluorescent imaging of brain slices,<br>National Instruments software for fiber photometry recording.
Data analysis	Observer XT 11 software software for behavior analysis,<br>QuPath 0.3.2 for fluorescent image analysis,<br>MATLAB for analyzing behavior and imaging data,<br>GraphPad Prism 9 for statistical analysis.

For manuscripts utilizing custom algorithms or software that are central to the research but not yet described in published literature, software must be made available to editors and reviewers. We strongly encourage code deposition in a community repository (e.g. GitHub). See the Nature Portfolio guidelines for submitting code & software for further information.

Data

Policy information about availability of data

All manuscripts must include a data availability statement. This statement should provide the following information, where applicable:

- Accession codes, unique identifiers, or web links for publicly available datasets
- A description of any restrictions on data availability
- For clinical datasets or third party data, please ensure that the statement adheres to our policy

All data that support the findings of this study are either present in figures and extended data, or available from the Dulac lab GitHub database (https://github.com/DulacLabHarvard/SocialNeed), or from the corresponding author upon request.

Research involving human participants, their data, or biological material

Policy information about studies with human participants or human data. See also policy information about sex, gender (identity/presentation), and sexual orientation and race, ethnicity and racism.

Reporting on sex and gender	*Use the terms sex (biological attribute) and gender (shaped by social and cultural circumstances) carefully in order to avoid confusing both terms. Indicate if findings apply to only one sex or gender; describe whether sex and gender were considered in study design; whether sex and/or gender was determined based on self-reporting or assigned and methods used. Provide in the source data disaggregated sex and gender data, where this information has been collected, and if consent has been obtained for sharing of individual-level data; provide overall numbers in this Reporting Summary. Please state if this information has not been collected. Report sex- and gender-based analyses where performed, justify reasons for lack of sex- and gender-based analysis.*
Reporting on race, ethnicity, or other socially relevant groupings	*Please specify the socially constructed or socially relevant categorization variable(s) used in your manuscript and explain why they were used. Please note that such variables should not be used as proxies for other socially constructed/relevant variables (for example, race or ethnicity should not be used as a proxy for socioeconomic status). Provide clear definitions of the relevant terms used, how they were provided (by the participants/respondents, the researchers, or third parties), and the method(s) used to classify people into the different categories (e.g. self-report, census or administrative data, social media data, etc.) Please provide details about how you controlled for confounding variables in your analyses.*
Population characteristics	*Describe the covariate-relevant population characteristics of the human research participants (e.g. age, genotypic information, past and current diagnosis and treatment categories). If you filled out the behavioural & social sciences study design questions and have nothing to add here, write "See above."*
Recruitment	*Describe how participants were recruited. Outline any potential self-selection bias or other biases that may be present and how these are likely to impact results.*
Ethics oversight	*Identify the organization(s) that approved the study protocol.*

Note that full information on the approval of the study protocol must also be provided in the manuscript.

Field-specific reporting

Please select the one below that is the best fit for your research. If you are not sure, read the appropriate sections before making your selection.

☒ Life sciences ☐ Behavioural & social sciences ☐ Ecological, evolutionary & environmental sciences

For a reference copy of the document with all sections, see nature.com/documents/nr-reporting-summary-flat.pdf

Life sciences study design

All studies must disclose on these points even when the disclosure is negative.

Sample size	The sample sizes for measuring social rebound in different mouse strains were determined by a power analysis using MATLAB command 'sampsizepwr' based on pilot experiments. In other experiments, the sample sizes were chosen based on common practices in animal behavior experiments (Wu et al 2014, Kohl et al 2018, Osterhout et al 2022) to reliably measure experimental effects and minimize the number of animals used in accordance with ethical guidelines.
Data exclusions	Animals in which the virus injection or lens implantation missed the target brain region, or weak or no viral expression was found, were excluded from analysis.
Replication	All the experiments were repeated at least 2-3 times with separate cohort of animals, and the reproducibility was confirmed.
Randomization	Animals were randomly assigned to experimental conditions or controls.
Blinding	Experimenters were blind to the mouse identity in Mrgprb4-lineage neuron lesion experiments. Due to obvious physical impairments

Blinding	observed in Nav1.8/DTA mice, we could not apply blind behavioral scoring in these mice. Quantification of cell numbers and marker co-expression was blinded to the investigator. Analysis of behavior, calcium and dopamine imaging data was automated by computer programs.

Reporting for specific materials, systems and methods

We require information from authors about some types of materials, experimental systems and methods used in many studies. Here, indicate whether each material, system or method listed is relevant to your study. If you are not sure if a list item applies to your research, read the appropriate section before selecting a response.

Materials & experimental systems

n/a	Involved in the study	
☐	☒	Antibodies
☒	☐	Eukaryotic cell lines
☒	☐	Palaeontology and archaeology
☐	☒	Animals and other organisms
☒	☐	Clinical data
☒	☐	Dual use research of concern
☒	☐	Plants

Methods

n/a	Involved in the study	
☒	☐	ChIP-seq
☒	☐	Flow cytometry
☒	☐	MRI-based neuroimaging

Antibodies

Antibodies used	Rabbit anti-c-Fos antibody (9F6, Cell Signaling); Alexa-568 anti-rabbit (Invitrogen, A11011)
Validation	Specificity of Rabbit anti-c-Fos antibody was validated using alternative c-Fos antibodies in tissue with known expression patterns. In addition, the manufacturers report that this antibody is specific to human, mouse and rat c-Fos protein and was validated by the manufacturers using western blot and ChIP analysis.

Animals and other research organisms

Policy information about studies involving animals; ARRIVE guidelines recommended for reporting animal research, and Sex and Gender in Research

Laboratory animals	Experiments were performed on female mice at 5-12 weeks, as detailed in the method section. The following mice were from the Jackson Laboratory: BALB/cJ (000651), DBA/2J (000671), C57BL/6J (000664), C3H/HeJ (000659), SWR/J (000689), FVB/NJ (001800), sighted FVB (FVB-Pde6b+, 004828), TRAP2 (also called Fos2A-iCreERT2, 030323), Ai9(RCL-tdT) (007909), Mc4r-2a-Cre (030759), Vglut2-IRES2-FlpO-D (030212), VGAT-2A-FlpO-D (029591), ROSA-DTA (009669), Nav1.8-Cre (036564). Pde6brd1 mutant mice in C57BL/6J background were from Dr. Connie Cepko (Harvard Medical School), and Mrgprb4-Cre mice from Dr. Ishmail Abdus-Saboor (Columbia University). Trpc2 knockout mice were generated previously by Dr. Dulac.
Wild animals	This study did not involve wild animals.
Reporting on sex	This study aims to study the fundamental drive for social interaction after social isolation, so all experiments were performed in female mice to avoid interfering behaviors such as aggression or mating seen in males.
Field-collected samples	This study did not involve field-collected samples.
Ethics oversight	All experiments were performed in accordance with NIH guidelines and approved by the Harvard University Institutional Animal Care and Use Committee (IACUC).

Note that full information on the approval of the study protocol must also be provided in the manuscript.
